# Learning English vocabulary from word cards: A research synthesis

**DOI:** 10.3389/fpsyg.2022.984211

**Published:** 2022-09-06

**Authors:** Yuanying Lei, Barry Lee Reynolds

**Affiliations:** ^1^Faculty of Education, University of Macau, Taipa, Macao SAR, China; ^2^Centre for Cognitive and Brain Sciences, University of Macau, Taipa, Macao SAR, China

**Keywords:** word cards, flashcards, vocabulary, receptive knowledge, productive knowledge

## Abstract

Researchers' interest in the learning of vocabulary from word cards has grown alongside the increasing number of studies published on this topic. While meta-analyses or systematic reviews have been previously performed, the types of word cards investigated, and the number of word card studies analyzed were limited. To address these issues, a research synthesis was conducted to provide an inclusive and comprehensive picture of how the use of word cards by learners results in vocabulary learning. A search of the Web of Science and Scopus databases resulted in 803 potential studies, of which 32 aligned with the inclusion criteria. Coding of these studies based on an extensive coding scheme found most studies assessed receptive vocabulary knowledge more often than productive vocabulary knowledge, and knowledge of vocabulary form and meaning were assessed more often than knowledge of vocabulary use. Results of effect size plots showed that more of the reviewed studies showed larger effects for the use of paper word cards than digital word cards, and for the use of ready-made word cards than self-constructed word cards. Results also indicated more studies showed larger effects for using word cards in an intentional learning condition compared with an incidental learning condition, and for using word cards in a massed learning condition compared with a spaced learning condition. Although a correlation was found between time spent using word cards and vocabulary learning outcomes, this correlation was not statistically significant. Learners that were more proficient in English learned more words from using word cards than those less proficient. These results suggest that future researchers should report learner proficiency, adopt reliable tests to assess vocabulary learning outcomes, compare the effectiveness of ready-made word cards and self-constructed word cards, and investigate the learning of different aspects of word knowledge. Teachers should provide learners guidance in how to use word cards and target word selection for self-construction of word cards. In addition, teachers should encourage learners to create word cards for incidentally encountered unknown words and use massed learning when initially working with these new words before using spaced learning for later retrieval practice.

## Introduction

Vocabulary knowledge is essential in second language (L2) learning (Barkat and Aminafshar, 2015; Reynolds and Shih, 2019). When learning English as a second language, acquiring vocabulary is “more important than mastering other language skills,” such as listening, speaking, reading, and writing (Lukas et al., 2020, p. 305). This is because vocabulary “acts as the foundation for learners to communicate” using the language (Lukas et al., 2020, p. 305). Learning a second language (L2) involves the learning of thousands of words (Laufer and Hulstijn, 2001; Nation, 2013a). In order to understand novels, newspapers, and spoken English, a vocabulary size of “3,000 to 4,000 word families” is needed (Nation, 2013a, p. 14). Researchers, teachers, and learners are interested in knowing the most direct route to learn so many words to be able to use language for these and other purposes.

Learners often engage in different activities and use different strategies to learn vocabulary. Vocabulary-learning activities are often compared to determine which activity is most effective. It is advantageous to learn vocabulary from word cards. For example, Webb et al. (2020, p. 16) suggested that word cards lead to “relatively large gains” in vocabulary knowledge compared to studying word lists. The strength of learning vocabulary from word cards comes from the fact that this activity is focused, efficient, and effective (Nation, 2013a). It is focused because “more attention can easily be paid to unknown words with the use of word cards” (Reynolds et al., 2020, p. 3). It is efficient because a large number of words “can be learned in a short time” using word cards (Nation, 2013a, p. 439). It is effective because word cards can be used for both “receptive and productive learning” (Nation and Webb, 2011, p. 41). Moreover, learners have been shown to prefer learning vocabulary from word cards compared to other vocabulary learning activities (e.g., Kuo and Ho, 2012). Therefore, word cards were chosen as the focus of the synthesis among a variety of vocabulary learning activities available to learners.

Although there is generally a consensus that learning vocabulary from word cards is advantageous, one must acknowledge other variables could enhance or reduce their effectiveness. Previous researchers have indicated that many variables affect vocabulary learning outcomes regardless of the vocabulary learning strategy employed by learners. For most intentional vocabulary learning strategies—including the use of word cards—these include how the strategy is employed (e.g., Uchihara et al., 2019) and language learner proficiency (e.g., Webb et al., 2020). More specifically for word card use, these include aspects of word knowledge (e.g., Nation, 2013a, Ch. 11) and types of word cards (e.g., Chen and Chan, 2019; Reynolds et al., 2020). Furthermore, word cards can only be effective when learners have been trained and understand how to use them (Reynolds et al., 2020). Therefore, these variables should be taken into consideration to understand whether word cards are effective for vocabulary learning.

It is worthwhile to conduct a synthesis of the word card literature to allow for generalization of the results reported in primary studies. A synthesis can help us to systematically review the word card literature, thereby providing a clearer picture of the overall effectiveness of word cards. Such a result can be useful for teachers, learners, and researchers, as a research synthesis can provide clear implications for research and teaching. Compared to meta-analysis, which “requires strict inclusion criteria for calculating effect sizes (ESs),” a research synthesis allows for “more varieties of relevant studies to be included” (Yang et al., 2021, p. 472). Therefore, this study gives a systematic and comprehensive review on the past research regarding vocabulary learning from word cards using a research synthesis methodology.

The current synthesis of primary empirical studies brings significance to the field of vocabulary learning from word cards for two main reasons. Firstly, there is growing interest in the effects that word cards have on vocabulary learning, evident through the large number of studies published on this topic. With this large body of research, it is not surprising that some existing meta-analyses and syntheses also touch on this topic. For example, Webb et al. (2020) conducted a meta-analysis to examine the effectiveness of many vocabulary learning activities including the use of word cards. As a meta-analysis requires some strict inclusion criteria, many relevant word card studies had to be eliminated. Similarly, several researchers have synthesized the word card literature. Unfortunately, their focus was on synthesizing the literature on one specific type of word cards rather than all types of word cards (Nakata, 2011; Lin and Lin, 2019; Ji and Aziz, 2021). Therefore, the previous meta-analyses and syntheses have not given an exclusive picture of how word cards lead to vocabulary learning. To fill this gap, this study adopts an inclusive synthesis approach to examine how the learners' use of word cards can lead to vocabulary learning.

Secondly, there are several potential variables that may affect vocabulary learning from word card use. For example, the effect of the use of digital word cards has been compared to paper word cards (e.g., Azabdaftari and Mozaheb, 2012; Chen and Chan, 2019). Some studies asked learners to self-construct word cards (e.g., Reynolds et al., 2020), while other studies provided word cards to learners (e.g., Oberg, 2011). However, it appears in the previous literature that researchers have not considered whether this could influence the effectiveness of word card use. The use of word cards is most often assumed to be an intentional vocabulary learning strategy. However, some researchers have reported to use word cards as an incidental learning strategy as well (e.g., Reynolds et al., 2020). Researchers have not considered whether the use of word cards is suitable for incidental learning. The literature usually suggests that learners use word cards in a spaced learning condition. However, some researchers have suggested learners to use massed learning as a large number of repeated encounters with the words will occur (Uchihara et al., 2019). Word cards were also reported to have been used for different amounts of time in previous studies (e.g., Webb et al., 2020). The amount of time spent learning from word cards might influence vocabulary learning. Moreover, most of the previous research involved learners at different levels of proficiency (e.g., Tan and Nicholson, 1997; Nakata, 2008). Different levels of language proficiency might result in varied amounts of vocabulary learning from word card use. In this regard, this study extends the discussion of learning L2 vocabulary through the use of word cards and includes potential variables that may affect the reported effects in the published word card literature.

Practically, the findings of this synthesis have the potential to benefit two stakeholders. Firstly, this study provides some suggestions for researchers who have been investigating vocabulary learning from word card use. The results can provide suggestions for a future research trajectory. Secondly, this study has the potential to provide teachers with advice on how they can incorporate the use of word cards into their classroom teaching and skill training for learners.

## Literature review

In this section, we review relevant vocabulary, word card, and theory literature before summarizing existing findings about the variables of interest to the present synthesis. Doing this helps to situate the research questions that follow. The results of this research synthesis builds on the literature that is covered in this section.

### Previous research syntheses on vocabulary learning from word cards

Previous meta-analysists and synthesists have conducted research related to English vocabulary learning activities and examined this field from different perspectives. For example, Webb et al. (2020) conducted a meta-analysis which focused on studies investigating “four types of intentional vocabulary learning activities, including flashcards, word lists, writing and fill-in-the-blanks” (Webb et al., 2020, p. 1). In their meta-analysis of 22 studies, Webb et al. (2020) found that “both flashcards and word lists led to relatively large gains in vocabulary knowledge while writing and fill-in-the-blanks lead to relatively small gains” (Webb et al., 2020, p. 19). However, their meta-analysis only included studies with treatments that lasted up to 1 day, i.e., studies with treatments that lasted longer than 1 day were excluded (Webb et al., 2020). Uchihara et al. (2019) conducted a meta-analysis which focused on the effects of repetition on incidental vocabulary learning. In their meta-analysis of 26 studies, Uchihara et al. (2019, p. 559) found that “there was a medium effect of repetition on incidental vocabulary learning.” However, their meta-analysis only included studies that adopted within participants design, i.e., studies that adopted between participants design were excluded. Various research designs deserve investigation, as there are an increasing number of empirical studies that have included separate groups with different interventions (Kose and Mede, 2018; Reynolds and Shih, 2019; Wulandari and Musfiroh, 2020). Both Webb et al.'s (2020) and Uchihara et al.'s (2019) meta-analyses focused on the form and meaning aspects of word knowledge. Other aspects of word knowledge should also be given attention by meta-analysists and synthesists. Other aspects of word knowledge require more rigid attention in vocabulary learning from word cards research (Uchihara et al., 2019), as vocabulary learning involves more than “associating the new words with their meaning” (Nakata, 2011, p. 20).

Nakata (2011) conducted a systematic review on digital word card programs for vocabulary learning. In this systematic review of 9 digital word card programs, Nakata (2011, p. 17) found that most digital word card programs “have been developed in a way that maximize vocabulary learning.” Lin and Lin (2019) conducted a systematic review and meta-analysis on vocabulary learning from digital word card use. In their systematic review and meta-analysis of 33 studies, Lin and Lin (2019) found that there was a positive and large effect of engagement in activities using digital word cards on vocabulary learning. Later, Ji and Aziz (2021) also conducted a systematic review of vocabulary learning from digital word card use. In their systematic review of 18 studies, Ji and Aziz (2021) also found that the use of digital word cards enhanced learners' vocabulary knowledge. These previous syntheses and meta-analyses gave insights on the effects of digital word card use but did not report on paper word card use or compare digital word cards to paper word cards. It is necessary to synthesize the studies that used digital word cards and paper word cards as it is important to see which type of word cards can result in better vocabulary learning outcomes.

Although previous syntheses have been investigating English language learning activities, few comprehensive syntheses have been conducted that focus on the use of word cards for vocabulary learning. Studies that utilized different research designs and assessed different aspects of word knowledge should be included for analysis, as the existing word card research was implemented in various research designs and assessed various aspects of word knowledge. In addition, various variables that might affect the vocabulary learning from word cards should be extracted from the studies for analysis.

It is evident that there is a growing interest in the effects that word cards have on vocabulary learning. This is shown from the number of different syntheses and meta-analyses that have been conducted on this topic (Nakata, 2011; Elgort, 2017; Lin and Lin, 2019; Kim and Webb, 2022). There is also a growing body of studies on word card use (Chen and Chan, 2019; Reynolds and Shih, 2019; Reynolds et al., 2020). However, the syntheses and the meta-analyses have not been very comprehensive in terms of the aspects of word knowledge assessed and the types of word cards used. The current synthesis is an attempt to give a systematic and comprehensive review on the past vocabulary learning research with a focus on word card use, hoping to provide some teaching implications and suggestions for future research.

### Theoretical perspectives of vocabulary learning from word cards

There are several theoretical perspectives that have been used to frame previous studies. However, the majority of studies have used the Involvement Load Hypothesis (Laufer and Hulstijn, 2001), the Pimsleur's Memory Schedule (Pimsleur, 1967), or the Dual-Coding Theory (Paivio, 1979). The word card studies included in the current synthesis relied on these theories for their research designs and interpretations of their results.

#### Involvement load hypothesis

The use of word cards is regarded as a task that has high involvement. The Involvement Load Hypothesis (ILH) is a “task-induced involvement” theory that consists of “three motivational and cognitive dimensions,” i.e., need, search, and evaluation (Laufer and Hulstijn, 2001, p. 2). Need is the “motivational, non-cognitive dimension of involvement” and refers to “whether unknown words are needed to complete a task” (Laufer and Hulstijn, 2001, p. 14; Yanagisawa and Webb, 2021, p. 489). Need is absent when an unknown word is not required (need is 0) (Yanagisawa and Webb, 2021). Need is moderate when it is “imposed by an external agent” (e.g., the learners are required to create word cards for teacher selected words) (need is 1), and it is strong when it is “imposed by the learners themselves” (e.g., the learners wish to create word cards for the incidentally encountered unknown words) (need is 2) (Laufer and Hulstijn, 2001, p. 14; Reynolds et al., 2020; Yanagisawa and Webb, 2021, p. 489). Search and evaluation are the “two cognitive dimensions of involvement” (Laufer and Hulstijn, 2001, p. 14). Search refers to the attempt to find an unknown L2 word's form or its meaning (Laufer and Hulstijn, 2001). Search is absent when the L2 word's form and its meaning are provided in a task (e.g., a reading comprehension task where new words are glossed) (search is 0) (Laufer and Hulstijn, 2001; Yanagisawa and Webb, 2021). Search is moderate when the learners need to find an unknown L2 word's form or its meaning using external resources (e.g., dictionaries or teachers) (search is 1), and it is strong when the learners need to engage in both receptive learning and productive learning (e.g., looking at the L2 word forms and trying to recall the L1 translations, and looking at the L1 translations and trying to recall the L2 word forms on word cards) (search is 2) (Reynolds et al., 2020; Yanagisawa and Webb, 2021). Evaluation involves “the comparison of a given word with other words” (Laufer and Hulstijn, 2001, p. 14). Evaluation is absent when the learners do not need to decide which word to use (evaluation is 0) (Yanagisawa and Webb, 2021). Evaluation is moderate when it entails recognizing differences between words with a context provided (e.g., a fill-in-the-blanks task with given words) (evaluation is 1), and it is strong when a word must be used in an authentic context (evaluation is 2) (e.g., a composition writing task using target words) (Laufer and Hulstijn, 2001; Yanagisawa and Webb, 2021). The strength of the involvement load can occur in any combination. ILH predicts that “higher involvement in a word induced by the task will result in better retention” (Laufer and Hulstijn, 2001, p. 20).

Some researchers used the ILH as a framework for designing their studies. For studies that used word cards, the involvement load was calculated as 6 out of a possible 6. For example, in Reynolds et al.'s (2020, p. 5) study, learners were required to “construct word cards for unknown words encountered while reading a class textbook.” In Reynolds et al.'s (2020, pp. 5–6) study, need was 2 (as the learners initiated the need to understand “the unknown words incidentally encountered during reading class texts”), search was 2 (as word cards were used for both receptive learning, i.e., the learners recalled the L1 translations by looking at the L2 word forms, and productive learning, i.e., the learners recalled the L2 word forms by looking at the L1 translations), and evaluation was 2 (as the learners compared “multiple meanings of the words” and used the chosen word to write a sentence on the word card). However, the ILH can only suggest the predictability of a task being useful or not for vocabulary learning. To address the issue of how memory works in the learning of vocabulary from word cards, the Pimsleur's Memory Schedule (Pimsleur, 1967) is more suitable.

#### Pimsleur's memory schedule

Previous researchers have suggested that the traditional way of memorizing words lacks scheduled repetition, which would lead to forgetting (Mondria and Mondria-De Vries, 1994). Repetition is “essential for vocabulary learning” in a foreign language (Nation, 2013a, p. 451). Pimsleur (1967) recommended “a memory schedule” which can be regarded as a guide “for determining the length of time that should occur between repetitions” (Kose and Mede, 2018, p. 5). Teachers can follow this schedule to space the recall of words previously learned by students. In this schedule, the rationale for determining the amount of time before recalling previously learned words is that most of the forgetting occurs after the initial learning of a word (Kose and Mede, 2018). This forgetting will slow down as time passes by if the words are periodically encountered (Kose and Mede, 2018). Pimsleur (1967) suggested how often new words should be repeated in order to keep them in a person's memory. It should be “5 s, 25 s (5^2^ = 25 s), 2 min (5^3^ = 125 s), 10 min (5^4^ = 625 s), and so on” (Nation, 2013a, p. 454). If learners are provided with opportunities for repetition of new words at the right time, their memories will be refreshed, and the retrieval of the words can improve retention.

Some researchers used the Pimsleur's Memory Schedule (PMS) (Pimsleur, 1967) as a framework for designing their studies. For example, Kose and Mede (2018) investigated the effects of vocabulary learning using digital word cards with a spaced repetition system following the PMS. By enabling learners to repeatedly be exposed to the target words at the right time, the learners in their study demonstrated a high level of vocabulary acquisition. This is because after the initial learning of a target word, the forgetting is very fast, but the forgetting on the second repetition will be slower (Nation, 2013a). Knowledge of vocabulary decreases less rapidly after each repetition of target words if the spacing has been increased (Mondria and Mondria-De Vries, 1994). However, most of the included studies used increased spacing rather than strictly following the PMS. However, if studies do not strictly use words cards only with printed text and instead opt for word cards containing pictorial elements, then the Dual-Coding Theory (Paivio, 1979) should be considered to understand how this added multimedia element affects learning.

#### Dual-coding theory

It is possible for the use of word cards to “combine visual and verbal information” to optimize “memorization of words” (Lavoie, 2016, p. 22). The Dual-Coding Theory (DCT) (Paivio, 1979) proposed that cognition occurs in two distinct codes, i.e., a verbal code for language, and a non-verbal code for mental imagery (Sadoski, 2006). When information is processed through two channels (verbal and non-verbal) instead of one, learners can “benefit from an additional or compensatory scaffold that supports L2 vocabulary learning” (Wong and Samudra, 2019, p. 1187). Visual representations of word meanings, such as pictures or multimedia, play an important role in vocabulary learning. In addition, written word forms must also be processed visually and learned as visible units (Sadoski, 2006). Therefore, dual coding of word cards might enhance memory recall of vocabulary.

Previous researchers have used the DCT as a framework for designing their studies. For example, in Lavoie's (2016) study, the experimental group that used word cards was compared to a control group. The word cards were presented with words and pictures to ensure the verbal and non-verbal information was processed at the same time. The results showed learners progressing in the learning of new words, demonstrating the additive effects of the two sources of input on vocabulary learning (Lavoie, 2016).

### Aspects of word knowledge

Vocabulary learning is not all or nothing. There are different aspects of word knowledge. At the most general level, vocabulary knowledge can be divided into three main categories, i.e., “form, meaning, and use” (Nation, 2013a, p. 48). Form refers to the “spoken form, written form and word parts”; meaning refers to “the connection between form and meaning, concepts, references and associations of a word”; and use refers to the “grammatical functions, collocations and constraints on use of a word” (Nation, 2013a, p. 539). Each of these aspects of word knowledge can be assessed productively or receptively. Receptive and productive vocabulary knowledge refers to the “learning direction” of vocabulary (Nation, 2013a, pp. 51–52). Productive knowledge of a word is what a learner “needs to know in order to use the word while speaking or writing,” while receptive knowledge is what a learner “needs to know to understand a word while reading or listening” (Crow, 1986, p. 242).

Table 1 (Nation, 2013a) lists these aspects of word knowledge, indicating which ones are well dealt with by learning form word cards, and which ones are partly dealt with by this strategy. Ideal learning occurs when vocabulary has been acquired both receptively and productively. Word cards “can be used for both receptive and productive learning” (Nation, 2013a, p. 441). For example, if the learners are using bilingual word cards with the “L1 on one side and the L2 on the other,” “looking at the L1 and trying to recall the L2 form” involves productive knowledge of form (Nation, 2013a, p. 446; Reynolds et al., 2020, p. 5). If the learners are “looking at the L2 and trying to recall the L1 meaning with the word cards,” it involves receptive knowledge of meaning (Reynolds et al., 2020, p. 5).

**Table 1:** Aspects of word knowledge dealt with by learning from word cards (Nation, 2013a, p. 442).

Form	Receptive	What does the word sound like?	✓
		What does the word look like?	✓✓
	Productive	How is the word written and spelled?	✓✓
Meaning	Receptive	What meaning does this word form signal?	✓✓
		What is included in the concept?	✓
	Productive	What word form can be used to express this meaning?	✓✓
Use	Receptive	In what patterns does the word occur?	✓
		What words or types of words must we use with this one?	✓
	Productive	In what patterns must we use this word?	✓
		What words or types of words must we use with this one?	✓

✓✓ = well dealt with, ✓ = partly dealt with.

### Variables that affect learning from word cards

Several variables have the potential of moderating the effectiveness of learning vocabulary from using word cards. These include the type of word cards used (i.e., paper or digital, ready-made or self-constructed), word cards used in different learning conditions (i.e., incidental or intentional, spaced or massed), period of time they are used, or if they are used by learners with different language proficiencies.

#### Paper and digital word cards

Paper word cards are defined as word cards made from paper-based materials (Nation, 2013a). The emergence of digital word cards allows learners to learn vocabulary on computers (Nakata, 2008) or mobile devices (Lai et al., 2020). The use of digital word cards can arouse learners' interest in vocabulary learning (Lin and Lin, 2019) and potentially lead to learning gains (Başoglu and Akdemir, 2010; Azabdaftari and Mozaheb, 2012; Tsai, 2018; Chen and Chan, 2019; Xodabande et al., 2021).

#### Ready-made and self-constructed word cards

Ready-made word cards, which are prepared by teachers or bought in stores, are common in the language learning classroom. For example, McDonald and Reynolds (2021) presented ready-made cards based on words taken from storybooks for learners. In addition to using ready-made word cards, learners can also acquire vocabulary by self-constructing their own word cards. For example, Reynolds et al. (2020) required learners to construct 10 word cards for each of the 10 readings in a textbook. Previous researchers have indicated that learners might have a strong affective bond with self-constructed word cards (Mondria and Mondria-De Vries, 1994). It is meaningful to know whether a learner should use self-constructed word cards or ready-made word cards. As learners may select the words by themselves for self-constructed word cards, this selection might affect their vocabulary learning.

#### Intentional and incidental learning conditions

The two broad approaches to vocabulary learning are intentional and incidental. Intentional vocabulary learning can be defined by whether learners know that “they will be tested on their vocabulary learning” (Webb et al., 2020, p. 2). If learners know of an “upcoming vocabulary test,” they may “pay special attention to vocabulary and engage in intentional learning” (Uchihara et al., 2019, p. 561). Incidental vocabulary learning is defined as “the learning that emerges through a meaning-focused comprehension task in which learners are not told of an upcoming vocabulary test” (Uchihara et al., 2019, p. 561). Thus, learners' awareness of a future assessment differentiates between incidental learning, where learners are “unaware of a subsequent vocabulary test,” and intentional learning, where “they know they will be tested” (Webb et al., 2020, p. 2).

#### Spaced and massed learning conditions

A massed learning condition refers to a learning condition in which words are repeated “during a single and continuous period of time,” while a spaced learning condition refers to a learning condition in which words are repeated “across a period of time at ever-increasing intervals” (Kose and Mede, 2018, p. 4). Spacing has often been operationalized “within a strictly controlled laboratory setting” in which learners study individual L2 words at different time intervals (Uchihara et al., 2019, p. 574). In this synthesis, the massed learning condition was operationalized as use of word cards within a single day, while the spaced learning condition was operationalized as use of word cards that lasted for more than 1 day (Uchihara et al., 2019). Previous researchers have examined the effect of spacing on vocabulary development. For example, Kuo and Ho (2012, p. 36) found a larger but non-significant effect on vocabulary learning when word cards were used in spaced learning conditions compared to massed learning conditions, because the effects of spaced learning might be reduced by retrieval activities in both learning conditions.

#### Time spent learning from word cards

Previous researchers were also interested in the amount of time that learners spent on learning from word cards (Webb et al., 2020). In Webb et al.'s (2020) meta-analysis of vocabulary learning activities, results showed that the number of minutes learners spent per word did not significantly influence vocabulary learning. In the present research synthesis, time spent learning from word cards was operationalized as the number of minutes the learners spent learning vocabulary using the cards.

#### Proficiency level of learners

The Common European Framework of Reference for Languages (CEFR) (Council of Europe, 2001) is “the most influential language framework in the field of second language teaching and assessment” (Fleckenstein et al., 2020, p. 2). “It describes foreign language competencies in three broad stages which can be divided into six proficiency levels,” i.e., A1/A2 for basic users, B1/B2 for independent users, and C1/C2 for proficient users (Fleckenstein et al., 2020, p. 2). Previous researchers have indicated that more advanced learners usually acquire more vocabulary than less proficient learners, as greater L2 knowledge should help learners to understand and use language (Webb et al., 2020).

### Testing vocabulary knowledge

In the previous research investigating the effects of word card use on vocabulary learning, researchers have used standardized tests and researcher-constructed tests. These tests have been used to assess different aspects of vocabulary knowledge (i.e., receptive and productive knowledge of form, meaning, and use). In this section, the standardized tests and the researcher-constructed tests used in these previous studies are introduced.

#### Standardized tests

Three main standardized tests have been used in the published literature. These include the Vocabulary Size Test (VST) (Nation and Beglar, 2007), the Updated Vocabulary Levels Test (UVLT) (Webb et al., 2017), and the New General Service Lists Test (NGSLT) (Browne et al., 2013). Table 2 provides example items from these standardized tests.

**Table 2:** Standardized tests of vocabulary knowledge.

**Type of test**	**Example item**
The Vocabulary Size Test (VST) (Nation and Beglar, 2007)	Maintain: Can they maintain it? a. keep it as it is b. make it larger c. get a better one than it d. get it
The Updated Vocabulary Levels Test (UVLT) (Webb et al., 2017)	___ formal and serious manner ___ winner of a sporting event ___ building where valuable objects are shown 1. bull 2. champion 3. dignity 4. hell 5. museum 6. solution
The New General Service Lists Test (NGSLT) (Browne et al., 2013)	Include: We are including it. a. paying b. changing c. adding d. reading

The VST (Nation and Beglar, 2007) was designed to measure a learner's overall English receptive vocabulary knowledge. It is one of the most popular tests used to measure vocabulary size. The VST consists of 140 multiple-choice items. It consists of “10 sampled target words from each of the 1,000-level word family” lists up to the 14,000 level extracted from the “100,000,000 token British National Corpus” (Reynolds et al., 2020, p. 4). Answering all items correctly indicates that the test taker knows the most frequent “14,000 word families” of English (Reynolds et al., 2020, pp. 4–5).

The UVLT (Webb et al., 2017) allows one to measure the mastery of vocabulary at different frequency levels. Specifically, the “first 1,000 most frequent words” of English to the “fifth 1,000 most frequent words” of English are assessed (Webb et al., 2017, p. 35). A test taker is presented with 30 questions per level. A test taker that scores “at least 26/30 (87%) has achieved mastery of that level” and might then focus on learning words from the next level (Webb et al., 2017, p. 56). However, the stricter criterion of 29/30 is recommended for masterly of the first three (1,000–3,000 word families) levels as those are commonly accepted as the basis for future vocabulary learning.

The NGSLT (Browne et al., 2013) is “a diagnostic instrument” designed to assess “written receptive knowledge” of the words on the New General Service List (NGSL) (Stoeckel et al., 2018, p. 5; Xodabande et al., 2021, p. 100). The NGSL is comprised of “2,800 high frequency words” and is designed to “provide maximal coverage of texts for learners of English” (Stoeckel et al., 2018, p. 5). The test is “a multiple-choice test that consists of 5 levels, each assessing knowledge of 20 randomly sampled words from a 560-word frequency based level of the NGSL” (Stoeckel et al., 2018, p. 5). The first level represents the most frequent words, the second level represents slightly less frequent words, and so forth. Answering correctly 16 or 17 items out of 20 indicates mastery of that level (Browne et al., 2013).

#### Researcher-constructed tests

Looking at “how well a particular word is known” is called measuring “depth of knowledge,” while looking at “how many words are known” is called measuring “breath of knowledge” (Nation, 2013a, p. 549). Table 3 (Nation, 2013a, p. 442) lists various aspects of what is involved in “knowing a word” and provides a corresponding example test item that has been used in previous research to assess that particular knowledge aspect.

**Table 3:** Researcher-constructed tests of vocabulary knowledge.

		**Study**	**Example item**
Form	Spoken	Samad and Makingkung (2020)	(P) Read aloud the word and spell it out loud.
	Written	Lukas et al. (2020)	(P) Name the pictures of animals correctly.
	Word parts	N/A	N/A
Meaning	Form and meaning	Fukushima (2019)	(R) Enter the L1 words after the displayed L2 English words. (P) Enter the L2 English words after the displayed L1 words.
	Concept and referents	N/A	N/A
	Associations	Oberg (2011, p. 136)	(R) Many KUT students ____ by bicycle, but some students take the train or bus. A. stick things together B. move around something C. hang out with friends D. insert into a slot E. commute to school F. spend money on G. recognize a face H. study mechanical engineering I. re-charge batteries J. play video games
Use	Grammatical functions	Alhuwaydi (2020)	(P) Specify the part of speech for words.
	Collocations	N/A	N/A
	Constraints on use	N/A	N/A

R, receptive knowledge; P, productive knowledge; N/A, No example item available in the included studies.

Previous word card research has assessed both receptive form knowledge and productive form knowledge. Receptive knowledge of form refers to whether a learner can recognize the “spoken form of a word, written form of a word, or the parts in a word” (Nation, 2013a, p. 538). Productive form refers to whether a learner can “pronounce a word correctly, spell and write a word, or produce appropriate inflected and derived forms of a word” (Nation, 2013a, p. 538). For example, Lukas et al. (2020) assessed the productive knowledge of form by having learners complete a word dictation task after they were provided a picture of an animal. Samad and Makingkung (2020) assessed the productive knowledge of form by having learners read aloud a word and spell it out loud.

Previous word card research has also assessed both receptive and productive word meaning. Receptive meaning refers to whether a learner can “recall the appropriate meaning for a word form, understand a range of uses of a word and its central concept, or recall common associations for a word” (Nation, 2013a, p. 538). Productive meaning refers to whether the learner can “produce an appropriate word form to express its meaning, use a word to refer to a range of items, or recall a word when presented with related ideas” (Nation, 2013a, p. 538). For example, Oberg (2011) assessed the receptive knowledge of meaning by having learners take a sentence fill-in-the-blank test (see Table 3). Fukushima (2019) assessed the receptive knowledge of meaning by asking learners to complete a test that required them to provide L1 Japanese for displayed L2 English words. Productive knowledge of meaning was also assessed by asking the learners to provide L2 English words after L1 Japanese words were displayed (Fukushima, 2019).

Previous word card research has assessed both receptive and productive knowledge of use. Receptive use refers to whether a learner can “recognize correct uses of a word in context, recognize appropriate collocations, or tell if a word is a common, formal, or infrequent word” (Nation, 2013a, p. 538). Productive use refers to whether a learner can “use a word in correct grammatical patterns, produce a word with appropriate collocations, or use a word at appropriate times” (Nation, 2013a, p. 538). For example, Alhuwaydi (2020) assessed the productive knowledge of use by asking learners to specify the part of speech for words.

### Purpose of the study

With a view to broadening our understanding of English vocabulary learning from word cards, this study attempts to provide an overview of relevant empirical studies to identity potential variables that may affect learning from word cards. The synthesis was guided by the following research questions (RQs):

RQ 1: What aspects of word knowledge have been investigated in the published word card research literature?
RQ 2: Which type of word cards has a larger effect on vocabulary learning? Digital or paper? Self-constructed or ready-made?
RQ 3: Which condition has a larger effect on vocabulary learning? Incidental or intentional? Spaced or massed?
RQ 4: What is the strength of the correlation between the time spent using word cards and vocabulary learning?
RQ 5: Which language proficiency group (basic, independent, and proficient) can learn the most vocabulary from using word cards?

## Methodology

### Research synthesis in language learning research

The primary goal of a research synthesis is to integrate empirical research findings by “drawing overall conclusions from many separate investigations that address identical or related hypotheses” (Cooper, 2017, pp. 170–171). The present study is a research synthesis of previous vocabulary learning studies which involve word card activities, attempting to provide generalizations of the practice of using word cards in L2 English learning and the effectiveness of word card usage in L2 English vocabulary development.

A well-designed research synthesis involves seven stages (Cooper, 2017, pp. 32–36): “(1) formulating the problem; (2) searching the literature; (3) gathering information from studies; (4) evaluating the quality of studies; (5) analyzing and integrating the outcomes of studies; (6) interpreting the evidence; and (7) presenting the results.” The seven stages for the current research synthesis are briefly summarized in this section and more detailed explanations are provided in the following sections. The first step is to formulate the problem. In this synthesis, after formulating the five research questions, the key concepts, constructs, and variables were clearly defined to distinguish relevant and irrelevant studies. The second step is searching the literature to identify relevant studies. To locate potential primary studies related to English vocabulary learning from word cards, a comprehensive literature search was conducted in the Web of Science (including SCI-Expanded, SSCI, AHCI, CPCI-S, CPCI-SSH, BKCI-S, BKCI-SSH, ESCI) and Scopus databases using search terms related to vocabulary and word cards. The third step is to gather information from studies. To identify the studies to include in the present research synthesis, a set of inclusion and exclusion criteria was applied to screen the retrieved studies after the literature search. Then, after applying the inclusion and exclusion criteria, a systematic coding process was applied using a coding scheme that helped identify important data for analysis. The reliability of this coding process was checked before further data analysis. The fourth step is to evaluate the quality of studies. In this synthesis, the Study Design and Implementation Assessment Device (Study DIAD) was used to evaluate the studies (Cooper, 2017). The fifth step is to analyze and integrate the outcomes of the primary studies. Results from the primary word card vocabulary learning studies were combined, identifying systematic data patterns regarding the practice of using word cards and its effects on vocabulary learning development (see Section Results). The sixth and seventh steps are to present and interpret the results. This was done through a discussion of this synthesis (see Section Discussion).

### Literature search

To locate potentially relevant studies on English vocabulary learning from word cards, the following electronic databases were comprehensively searched: Scopus and Web of Science (WOS) (including Science Citation Index Expanded, Social Sciences Citation Index, Arts and Humanities Citation Index, Conference Proceedings Citation Index—Science, Conference Proceedings Citation Index—Social Science and Humanities, Book Citation Index—Science, Book Citation Index—Social Sciences and Humanities, Emerging Sources Citation Index). The search covered all document types including journal articles, conference papers, and book chapters. The literature search covered the period from 1945 to July 2021. There were no limits on the publication period for the included studies. All studies were searched and screened for inclusion within the databases, in order to be as inclusive as possible.

The key terms related to vocabulary and word cards were searched in the databases by title, yielding 803 results. A set of inclusion and exclusion criteria were applied to determine the studies to be included in the synthesis (see Inclusion and Exclusion Criteria). Duplicated studies were removed. The combinations of search terms and Boolean operators (“AND” or “OR”) used in the database searches are presented in Table 4.

**Table 4:** The key terms used in the database searches.

Search Terms for Vocabulary	*word* OR vocab* OR collocation* OR “n gram*” OR idiom* OR lexic* OR lexeme* OR “lexical bundle*” OR chunk* OR phras* OR pattern* OR formulaic* OR figurative* OR fixed-frame* OR binomial*
	AND
Search Terms for Word Cards	wordcard* OR flashcard* OR “word card” OR “word cards” OR card OR cards

### Inclusion and exclusion criteria

After the initially eligible studies for the synthesis were identified, they were carefully examined based on a set of inclusion and exclusion criteria. Studies that were based on empirical data were included. Besides quantitative empirical studies, qualitative empirical studies were also included in this synthesis. In addition, studies that were written in English were retrieved in this synthesis, as the published studies on international and English-language journals or conferences were generally regarded as quality studies. Studies with the following features were included:

1. The study was based on empirical data.
2. The study was written in English.

Studies with the following features were excluded:

1. The study was not related to language learning.
2. The study measured non-English language outcomes.
3. The study was not related to vocabulary.
4. The study participants were non-mainstream learners.
5. The study participants were native English speakers.

**Figure 1:**
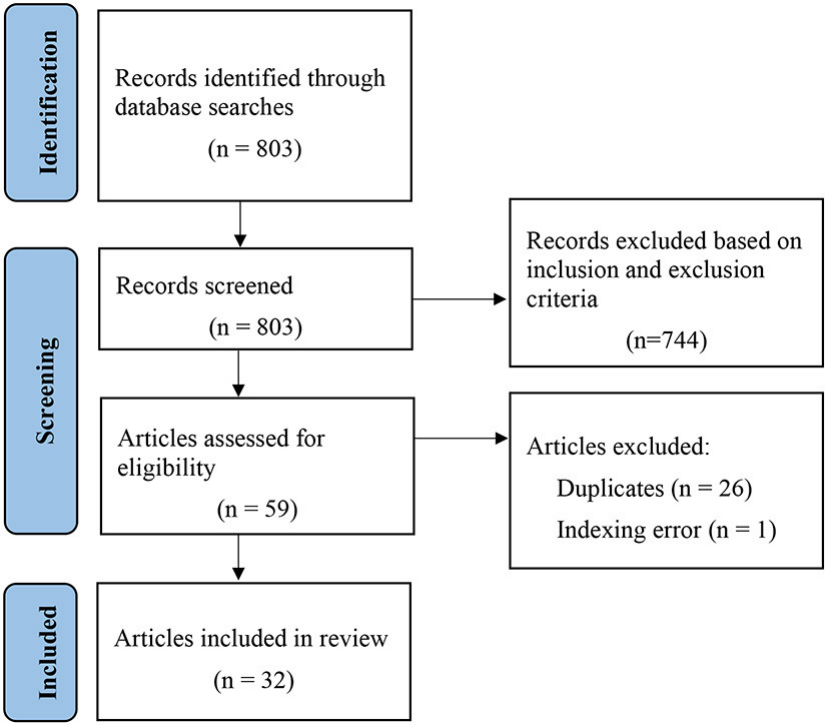
PRISMA flow diagram.

The 803 studies potentially eligible for the synthesis were then reviewed carefully to identify relevant studies based on the inclusion and exclusion criteria. The titles, abstracts, and full texts (when necessary) of all retrieved papers were reviewed. Seven hundred and forty four were excluded based on the inclusion and exclusion criteria, 26 duplicates were excluded, and 1 was removed due to an indexing error. Overall, the search yielded a sample of 32 studies that were included in the research synthesis. The corresponding full-text documents were obtained. The database search process is presented in a Preferred Reporting Items for Systematic Reviews and Meta-Analyses (PRISMA) flow diagram (Page et al., 2021) in Figure 1.

### Coding

While the content of every research synthesis coding guide will be unique to the research questions asked, there are certain broad types of information that every synthesist will want to gather from primary research reports (Cooper, 2017). The information to include on a coding guide is classified into eight categories: “(1) the report; (2) the predictor or independent variables; (3) the setting in which the study took place; (4) participant and sample characteristics; (5) the dependent or outcome variables and how they were measured; (6) the type of research design; (7) statistical outcomes and effect sizes; and (8) coder and coding process characteristics” (Cooper, 2017, p. 120). In addition, previous research syntheses on vocabulary learning from word cards coded various variables, including proficiency level and educational level of learners (Webb et al., 2020), number of target words (Wright and Cervetti, 2017; Webb et al., 2020), test timing (Webb et al., 2020) and spacing (Uchihara et al., 2019). Therefore, after the 32 studies that met the inclusion criteria were identified, Cooper's (2017) coding suggestions and the coded variables of previous research syntheses involving word cards were used to develop the coding scheme for this synthesis.

Specifically, this resulted in seven coding categories: (a) bibliographic information (e.g., author, year of publication), (b) learner characteristics (e.g., sample size, proficiency level), (c) word card characteristics (e.g., origin of word cards, digital integration), (d) methodological characteristics (e.g., study design, theoretical perspective), (e) learning conditions (e.g., spacing), (f) aspects of word knowledge (e.g., receptive form, productive form), and (g) results (e.g., mean of experimental group posttest scores). Supplementary Table 1 provides a detailed description of the coding scheme.

Some of the data was not available in the retrieved research, so the authors of the studies were contacted to request this information. Additional information was gratefully received from four authors (Kose and Mede, 2018; Alhuwaydi, 2020; Hidayat and Yulianti, 2020; Xodabande et al., 2021).

To establish the reliability of the coding procedures, 5 studies (15.63%) were randomly selected and independently coded by a researcher familiar with the process of a research synthesis. Following Boulton and Cobb's (2017) approach, the inter rater reliability was assessed by counting the number of discrepancies between the two researchers' coding. The agreement was found to be 75%. Then, a discussion about the discrepancies was conducted with the researcher. Another 5 studies (15.63%) were randomly selected from the remaining 27 studies and independently coded, then the agreement was found to be 93%. Any remaining disagreements were satisfactorily resolved through discussion, and the coding book was refined where necessary.

### Evaluation of included studies

The Study Design and Implementation Assessment Device (Study DIAD) (Cooper, 2017) was applied to evaluate the included studies. Studies were evaluated with the following four global questions in the Study DIAD (Cooper, 2017, pp. 170–171):

1. Fit Between Concepts and Operations: Were the participants in the study treated and the outcomes measured in a way that is consistent with the definition of the intervention and its proposed effects?
2. Clarity of Casual Inference: Did the research design permit an unambiguous conclusion about the intervention's effectiveness?
3. Generality of Findings: Was the intervention tested on participants, settings, outcomes, and occasions representative of its intended beneficiaries?
4. Precision of Outcome Estimation: Could accurate estimates of the intervention's impact be derived from the study report?”

After detailed evaluation, all 32 included studies were verified to be quality studies.

## Results

### Overview of primary studies

This section provides an overview of the 32 primary studies. Supplementary Table 2 presents the detailed information of these studies in chronological order. Bibliographic information, learner characteristics, word card characteristics, methodological characteristics and learning conditions are explained below.

### Bibliographic information

In terms of the type of publication, 81% of the studies were journal articles (*k* = 26), followed by conference papers (*k* = 4) and a book chapter (*k* = 1). In terms of the country or region, 25% of the studies were from Taiwan (*k* = 8), 25% were from Iran (*k* = 8), followed by Indonesia (*k* = 4), Turkey (*k* = 4), Japan (*k* = 3), Malaysia (*k* = 1), Macau (*k* = 1), Canada (*k* = 1), New Zealand (*k* = 1), and Saudi Arabia (*k* = 1).

**Figure 2:**
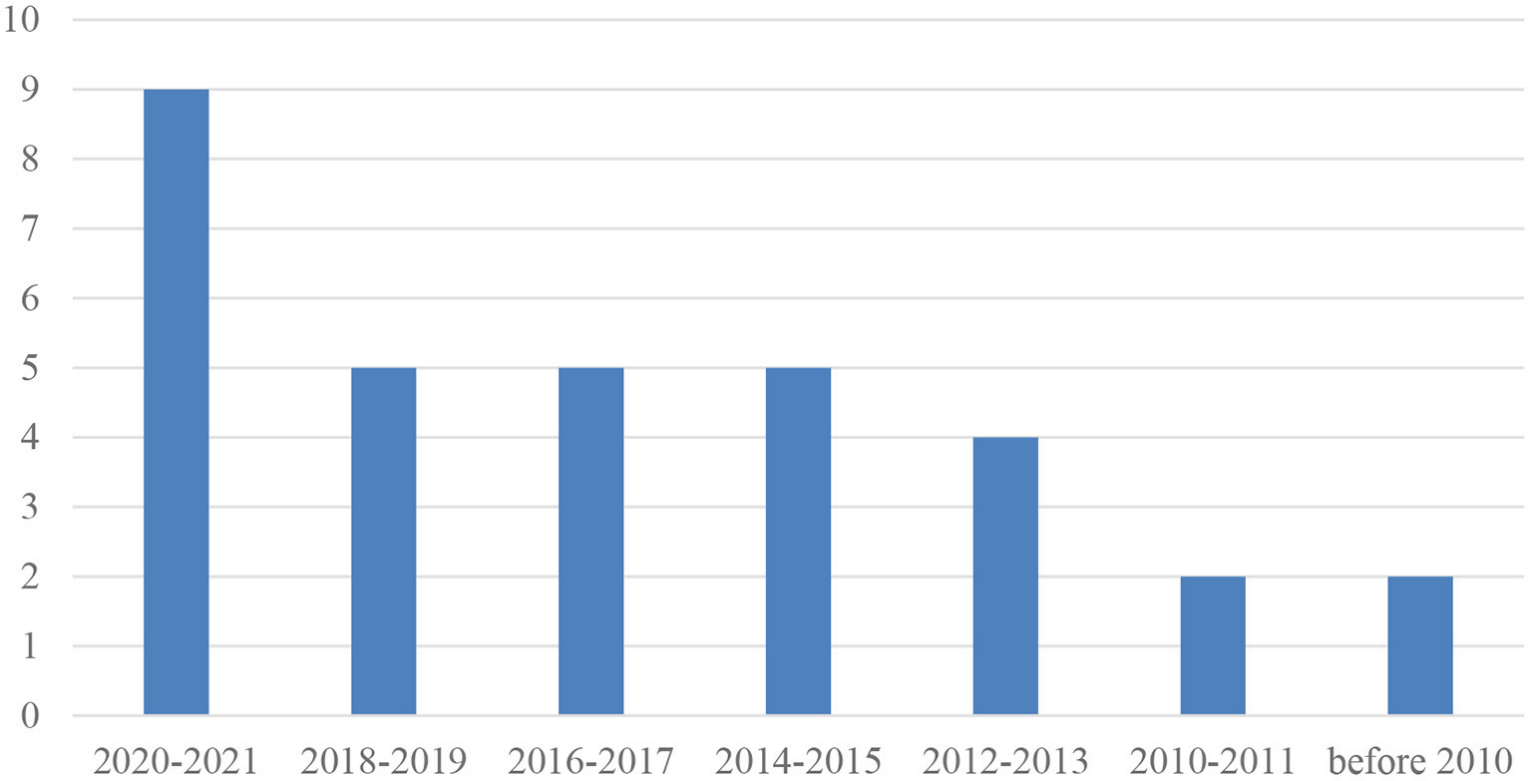
Frequency of year of publication.

The frequency of year of publication is reported in Figure 2. Although the literature search covered the period from 1945 to July 2021, there was only one study (Tan and Nicholson, 1997) from the 1990s that met the inclusion criteria for the synthesis. Thirty one out of the 32 studies were published from 2008 to 2021. As shown in Figure 2, 9 studies were published between 2020 and 2021, 5 studies between 2018 and 2019, 4 studies between 2016 and 2017, 6 studies between 2014 and 2015, 4 studies between 2012 and 2013, 2 studies between 2010 and 2011, and 2 studies before 2010.

### Learner characteristics

In terms of the proficiency level, 53% (*k* = 17) of the studies reported the proficiency level of the learners. Among the 32 studies, 25% of the studies involved learners at the B1 level (*k* = 8), 12.5% at the A1 level (*k* = 4), followed by B2 (*k* = 3), A2 (*k* = 1), C1 (*k* = 1), and none at the C2 level (*k* = 0). 46.88% did not report this data (*k* = 15). In terms of the educational level, 93.75% (*k* = 30) of the studies reported the educational level of the learners. Among the 32 studies, 31.25% of the studies involved learners at the university level (*k* = 10), 28.13% at the secondary level (*k* = 9), 21.88% at the primary level (*k* = 7), and 15.63% at the preprimary level (*k* = 5). 6.25% did not report this data (*k* = 2). With respect to L1 backgrounds, 88% (*k* = 28) of the studies reported this data. Among these 32 studies, 25% recruited L1 Mandarin learners (*k* = 8), followed by Turkish (*k* = 5), Indonesian (*k* = 4), Japanese (*k* = 3), Farsi (*k* = 3), Malaysian (*k* = 1), Cantonese (*k* = 1), French (*k* = 1), Persian (*k* = 1), and Arabic (*k* = 1). 12.5% did not report this data (*k* = 4).

### Word card characteristics

In terms of the origin of word cards, 81.25% of the studies used ready-made word cards (*k* = 26), and 18.75% used self-constructed word cards (*k* = 6). In terms of the digital integration, 40.63% of the studies did not use digital word cards (*k* = 13), 31.25% integrated used a computer program with word cards (*k* = 10), and 28.13% used a mobile app with word cards (*k* = 9). In terms of the semantic relatedness, 21.88% of the studies reported the data (*k* = 7). Among the 32 studies, 18.75% of the studies used semantic clustering of words (*k* = 6), 3.13% used thematic clustering of words (*k* = 1), and 78.13% did not report this data (*k* = 25). With respect to the type of assessed vocabulary, 69% of the studies reported the data. Among the 32 studies, 46.88% assessed specific vocabulary knowledge (*k* = 15), 21.88% assessed general vocabulary knowledge (*k* = 7), and 31.25% did not report this data (*k* = 10). In terms of the type of vocabulary test used in previous studies, 90.63% used researcher-constructed tests (*k* = 29), followed by VST (*k* = 2), VLT (*k* = 1), and NGSLT (*k* = 1).

### Methodological characteristics

In terms of the study design, 71.88% of the studies used an independent-group pretest-posttest design (*k* = 23), 18.75% used a single-group pretest-posttest design (*k* = 6), followed by other designs (*k* = 3). In terms of the theoretical perspectives, only 9.38% of the studies reported the use of any theoretical perspective (*k* = 3) for framing the studies. These perspectives included the Involvement Load Hypothesis (*k* = 1) (Laufer and Hulstijn, 2001), the Pimsleur's Memory Schedule (*k* = 1) (Pimsleur, 1967), and the Dual-Coding Theory (*k* = 1) (Paivio, 1979). With respect to a control group, 34.38% of the studies had a control group (*k* = 11), and 66.63% did not have a control group (*k* = 21). In terms of the pretest use, 87.5% conducted a pretest (*k* = 28) and 12.5% did not (*k* = 4). With respect to the test timing, 100% conducted an immediate posttest (*k* = 32), 25% of the studies conducted a delayed posttest (*k* = 8), and 25% conducted both (*k* = 8).

### Learning conditions

In terms of the approaches, 96.88% of the studies applied intentional learning (*k* = 31), and 6.25% applied incidental learning (*k* = 2). With respect to the spacing, 81.25% of the studies reported the data (*k* = 26). Among the 32 studies, 75% asked learners to apply spaced learning (*k* = 24), while 9.38% had learners apply massed learning (*k* = 3), and 3.13% investigated both conditions (*k* = 1). 18.75% did not report this data (*k* = 6).

### Calculation of effect sizes (ESs)

The studies included in the synthesis were not conducted with identical research designs, i.e., the included studies could be single-group pretest-posttest design or independent-groups pretest-posttest design. Due to the discrepancies in the designs, the guidelines suggested by Morris and DeShon (2002, pp. 107–108) and Navarro (2013, p. 382) were followed to calculate the ESs, as described below.

1. In the single-group pretest-posttest design (formula 1):

$$\text{ES}=\frac{{\text{Mean}}_{\text{post},\text{ E}}{-\text{ Mean}}_{\text{pre},\text{ E}}}{\surd ({\text{SD}}_{\text{post},\text{ E}}^{\text{2}}{+\text{ SD}}_{\text{pre},\text{ E}}^{\text{2}})}$$

2. In the independent-groups pretest-posttest design (formula 2):

$$\begin{array}{l} \text{ES}=\frac{\text{Mea}{\text{n}}_{\text{post},\text{ E}}-\text{Mea}{\text{n}}_{\text{pre},\text{E}}}{\text{S}{\text{D}}_{\text{pre},\text{ E}}}-\frac{\text{Mea}{\text{n}}_{\text{post},\text{ C}}-\text{Mea}{\text{n}}_{\text{pre},\text{C}}}{\text{S}{\text{D}}_{\text{pre},\text{ C}}} \end{array}$$

3. In the independent-groups pretest-posttest design (formula 3; when mean and standard deviation of pretest scores are not available in the published literature):

$$\text{ES}=\frac{{\text{Mean}}_{\text{post},\text{ E}}{-\text{ Mean}}_{\text{post},\text{C}}}{({\text{SD}}_{\text{post},\text{ E}}{+\text{ SD}}_{\text{post},\text{ C}})/2}$$

In the above formulas, post = posttest; pre = pretest; E = experimental group; C = control group; SD = standard deviation. In terms of formula 2 and 3, for independent-groups pretest-posttest studies that did not include a control group (e.g., only included two or more experimental groups), an experimental group, i.e., the least interfering experimental group, was treated as a control group. For example, Barkat and Aminafshar's (2015) study did not include a control group. In their study, learners were assigned into three experimental groups, i.e., paper word cards group, digital word cards group, as well as paper and digital word cards group. In this synthesis, only the first two experimental groups in their study were analyzed, and the second experimental group was treated as a control group.

It has been recommended by researchers that one study should ideally provide only one ES (Light and Pillemer, 1984). As all included studies conducted an immediate posttest, only immediate posttest scores rather than delayed posttest scores were extracted for ES calculation in each study. It should also be mentioned that the included studies did not assess identical aspects of word knowledge. However, the word knowledge was assessed sequentially in most studies. To calculate the ESs, the data of the first receptive knowledge assessment was extracted from each study to prevent practice effects (i.e., the previous test could affect the subsequent test performance) and gather unified data.

Specifically, 78.13% of the studies (*k* = 25) provided means and standard deviations needed for the computation of effect sizes in this synthesis. Conservative estimates of ESs were filled in for the remaining studies (*k* = 7) that had missing data, i.e., assigning ESs of zero, as minimum treatment effect was assumed (Light and Pillemer, 1984).

When the ESs were calculated, an effect direction plot, i.e., a visual display of non-standardized effects across included studies, was then generated (Thomson and Thomas, 2013). In addition, an effect size plot was constructed, i.e., ESs were categorized by their size and visually presented. This synthesis method was utilized to answer RQ 2, 3, and 5, which, respectively, concern the type of word cards used, word cards used in different learning conditions, and word cards used by learners with different language proficiencies.

### Research question 1: What aspects of word knowledge have been investigated in the published word card research literature?

The first research question concerns the aspects of word knowledge investigated in the previous studies. To examine what aspects of word knowledge were investigated by researchers, 29 out of the 32 studies (91%) which indicated the aspect of word knowledge assessed were included for analysis (Tan and Nicholson, 1997; Nakata, 2008; Başoglu and Akdemir, 2010; Oberg, 2011; Azabdaftari and Mozaheb, 2012; Komachali and Khodareza, 2012; Kuo and Ho, 2012; Chien, 2013, 2015; Nikoopour and Kazemi, 2014; Barkat and Aminafshar, 2015; Hamzehbagi and Bonyadi, 2015; Özer and Koçoglu, 2015; Galedari and Basiroo, 2016; Lavoie, 2016; Aminafshar, 2017; Saputri, 2017; Wu et al., 2017; Chen and Chan, 2019; Fukushima, 2019; Reynolds and Shih, 2019; Alhuwaydi, 2020; Hidayat and Yulianti, 2020; Lukas et al., 2020; Samad and Makingkung, 2020; Wulandari and Musfiroh, 2020; Yüksel et al., 2020; Xodabande et al., 2021).

**Figure 3:**
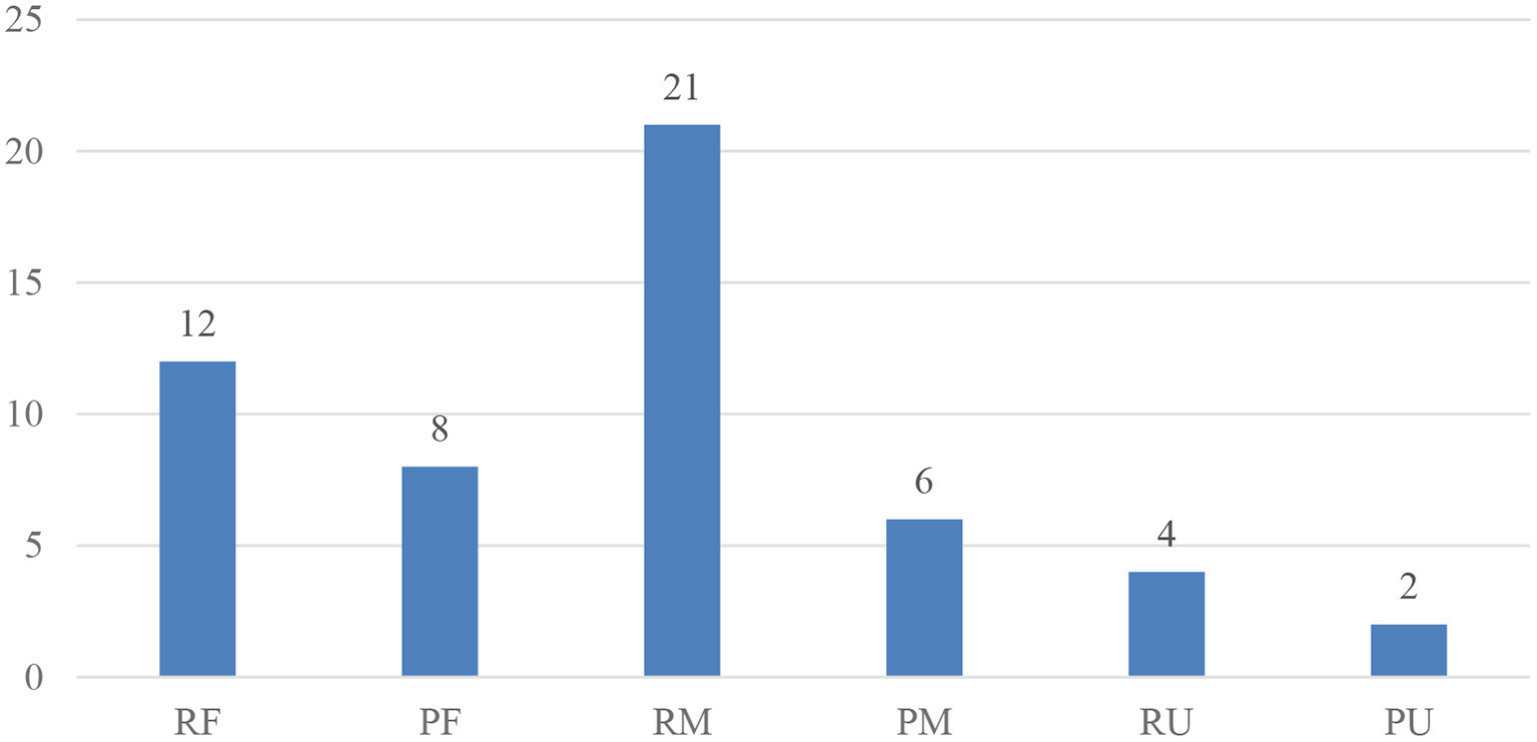
Frequency of word knowledge type (*k* = 29). Max = 29 as only 29 of the 32 studies provided necessary data. RF, receptive knowledge of form; PF, productive knowledge of form; RM, receptive knowledge of meaning; PM, productive knowledge of meaning; RU, receptive knowledge of use; PU, productive knowledge of use. As more than one aspect of word knowledge might have been assessed within a single study, the total is higher than 29.

Firstly, the aspects of word knowledge assessed in each of the 29 studies were coded. Secondly, the frequency of studies that assessed each aspect of word knowledge (i.e., how many studies out of the 29 studies investigated the different types of word knowledge) was calculated. Results are presented in Figure 3. In these 29 studies, 72.41% assessed receptive knowledge of meaning (RM) (*k* = 21), 41.38% assessed receptive knowledge of form (RF) (*k* = 12), 27.59% assessed productive knowledge of form (PF) (*k* = 8), 20.69% assessed productive knowledge of meaning (PM) (*k* = 6), 13.79% assessed receptive knowledge of use (RU) (*k* = 4), and 6.90% assessed productive knowledge of use (PU) (*k* = 2).

Based on the frequency of studies that assessed each aspect of word knowledge, most studies assessed receptive vocabulary knowledge more often than productive vocabulary knowledge. In addition, knowledge of vocabulary form and meaning were assessed more often than knowledge of vocabulary use.

### Research question 2: Which type of word cards has a larger effect on vocabulary learning? Digital or paper? Self-constructed or ready-made?

The second research question concerns the types of word cards that were used in the previous studies. To examine the effects of word card type on vocabulary learning, all 32 studies that provided the necessary data were analyzed. The digital integration and the origin of word cards for each of the 32 studies were coded and their ESs were calculated.

Firstly, results concerning the digital integration showed that among the studies, 50% of the studies used paper word cards that did not contain digital integration (*k* = 16), 31.25% used digital word cards in a computer program (*k* = 10), and 28.13% used digital word cards in a mobile app (*k* = 9). Digital word cards (59.38%, *k* = 19) were used more often by researchers than paper word cards (50%, *k* = 16). Specifically, digital word cards in a computer program (31.25%, *k* = 10) were used more often than digital word cards in a mobile app (28.13%, *k* = 9).

Secondly, results concerning the origin of the word cards showed that among the studies, 81.25% used ready-made word cards (*k* = 26), and 18.75% used self-constructed word cards (*k* = 6). Among the two word card types, ready-made word cards were used more often by researchers than self-constructed word cards.

Thirdly, the effect direction plot was constructed and visually presented in Table 5. Arrows were used to indicate reported effect direction (positive effect ▲, negative effect ▼, or no change ◀▶) (Thomson and Thomas, 2013). Arrows were also used to indicate sample size (large arrow=sample size equals or >50, small arrow=sample size smaller than 50) (Thomson and Thomas, 2013). Among the 32 studies, 75% reported a positive effect (*k* = 24) and 25% reported no change (*k* = 8). There was a trend showing most studies that applied the word cards, either digital or paper, ready-made or self-constructed, showed a positive effect on vocabulary learning.

**Table 5:** Effect direction plot for type of word cards (*k* = 32).

**Studies**	**Sample size (E/C)**	**Digital integration**	**Origin of word cards**	**Outcomes**
Tan and Nicholson (1997)	42	Paper	Ready-made	▴
Nakata (2008)	67/74#	Digital (computer program)	Ready-made	▲
Başoglu and Akdemir (2010)	29/29	Paper and digital (mobile app)	Ready-made	▴
Oberg (2011)	28/36#	Digital (computer program)	Ready-made	▴
Azabdaftari and Mozaheb (2012)	40/40#	Paper and digital (mobile app)	Ready-made	▴
Komachali and Khodareza (2012)	25/25	Paper	Ready-made	▴
Kuo and Ho (2012)	30/30#	Paper	Ready-made	▴
Chien (2013)	76	Digital (computer program)	Self-constructed	◀▶
Nikoopour and Kazemi (2014)	109	Digital (mobile app)	Ready-made	◀▶
Barkat and Aminafshar (2015)	15/15#	Digital (computer program)	Ready-made	▴
Chien (2015)	64	Digital (computer program)	Self-constructed	◂▸
Hamzehbagi and Bonyadi (2015)	30/30	Paper	Ready-made	▴
Lavoie (2016)	39/15	Paper	Ready-made	▴
Özer and Koçoglu (2015)	89	Digital (computer program)	Ready-made	◀▶
Galedari and Basiroo (2016)	30/30	Paper	Ready-made	▴
Aminafshar (2017)	15	Digital (computer program)	Ready-made	◂▸
Saputri (2017)	13	Paper	Ready-made	▴
Wu et al. (2017)	10/10	Paper	Ready-made	▴
Kose and Mede (2018)	17/17	Digital (mobile app)	Self-constructed	▴
Tsai (2018)	9/9#	Paper and digital (mobile app)	Ready-made	▴
Chen and Chan (2019)	48/50	Paper and digital (computer program)	Ready-made	▴
Fukushima (2019)	30	Digital (mobile app)	Ready-made	▴
Reynolds and Shih (2019)	100	Paper	Self-constructed	▲
Alhuwaydi (2020)	42	Digital (mobile app)	Self-constructed	▴
Hidayat and Yulianti (2020)	27/26#	Digital (computer program)	Ready-made	◂▸
Lai et al. (2020)	38/20	Digital (mobile app)	Ready-made	▴
Lukas et al. (2020)	52	Paper	Ready-made	▲
Reynolds et al. (2020)	50	Paper	Self-constructed	▲
Samad and Makingkung (2020)	20	Paper	Ready-made	◂▸
Wulandari and Musfiroh (2020)	34/33	Paper	Ready-made	▴
Yüksel et al. (2020)	57	Digital (computer program)	Ready-made	▲
Xodabande et al. (2021)	36/19	Paper and digital (mobile app)	Ready-made	▴

E, experimental group; C, control group. # = an experimental group, i.e., the least interfering experimental group was treated as a control group. ▴ or ▲ = positive effect, ▾ = negative effect, ◀▶ or ◂▸ = no change. Large arrow = sample size equals or >50, small arrow = sample size smaller than 50.

**Figure 4:**
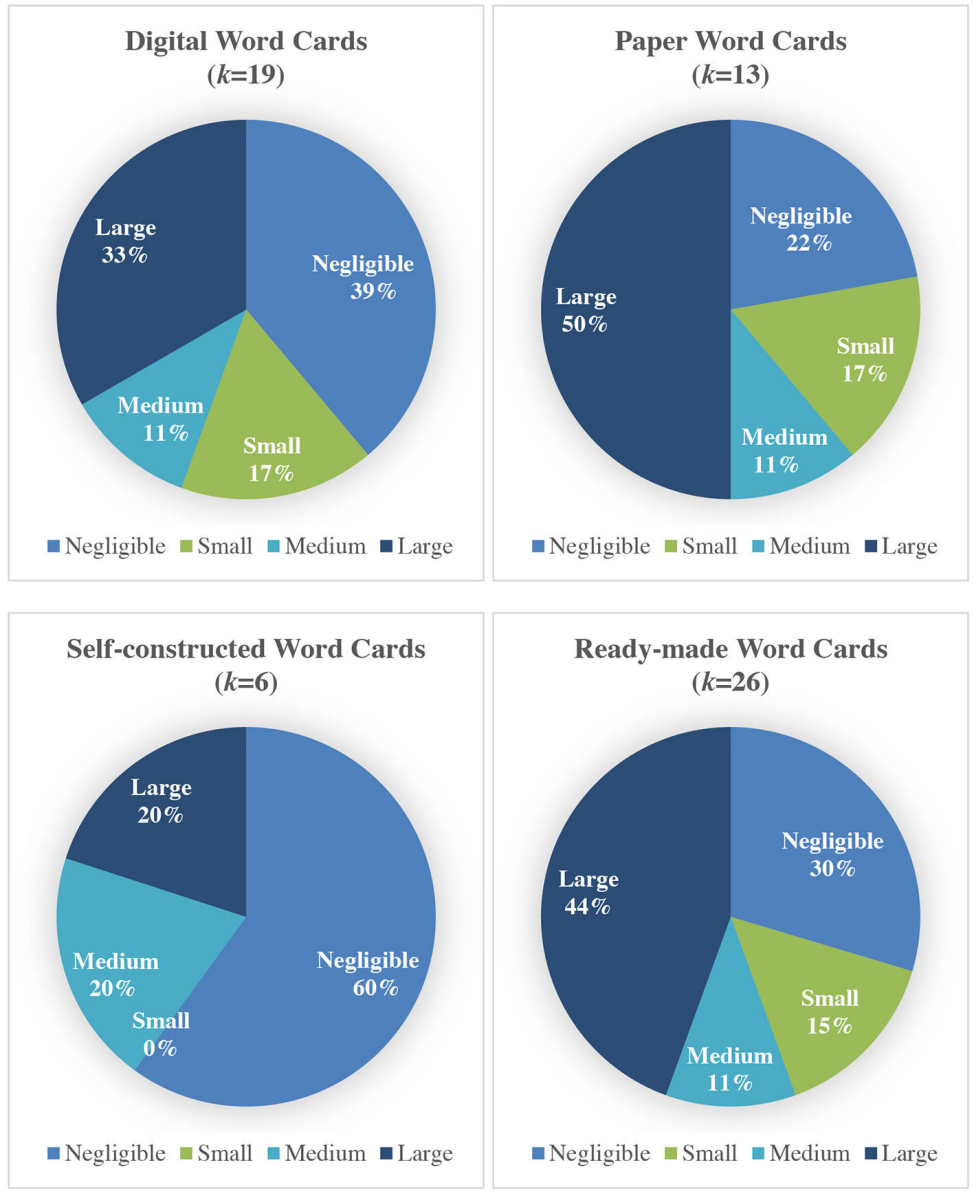
Effect size plot for type of word cards (*k* = 32).

Lastly, the effect size plot was constructed, i.e., ESs were categorized by their size and visually presented in Figure 4. ESs for these studies were interpreted according to Cohen's guidelines, i.e., < .2 is negligible, .2 is small, .5 is medium, and .8 is large (Larson-Hall, 2010). In terms of digital integration, studies that used digital word cards showed varied effects, i.e., small (17%), medium (11%) and large effects (33%). Studies that used paper word cards also showed small (17%), medium (11%) and large effects (50%). In terms of the origin of the word cards, studies that used self-constructed word cards showed medium (20%) and large (20%) effects. Studies that used ready-made word cards showed small (15%), medium (11%) and large (44%) effects.

Based on the results of the effect size plot, more of the reviewed studies showed a larger effect for the use of paper word cards compared to digital word cards. In addition, more of the reviewed studies showed a larger effect for the use of ready-made word cards than self-constructed word cards.

### Research question 3: Which condition has a larger effect on vocabulary learning? Incidental or intentional? Spaced or massed?

The third research question concerns the learning conditions that were applied in the previous studies. To examine the effects of learning conditions on vocabulary learning, all 32 studies that provided the data on the approach (i.e., incidental or intentional learning condition) were analyzed, then 26 studies (81%) that provided the data on spacing (i.e., spaced or massed learning condition) were analyzed (Nakata, 2008; Başoglu and Akdemir, 2010; Azabdaftari and Mozaheb, 2012; Komachali and Khodareza, 2012; Kuo and Ho, 2012; Chien, 2013, 2015; Nikoopour and Kazemi, 2014; Barkat and Aminafshar, 2015; Özer and Koçoglu, 2015; Galedari and Basiroo, 2016; Lavoie, 2016; Aminafshar, 2017; Saputri, 2017; Wu et al., 2017; Kose and Mede, 2018; Tsai, 2018; Chen and Chan, 2019; Fukushima, 2019; Reynolds and Shih, 2019; Hidayat and Yulianti, 2020; Lai et al., 2020; Lukas et al., 2020; Reynolds et al., 2020; Yüksel et al., 2020; Xodabande et al., 2021). The learning approach (i.e., incidental learning or intentional learning) and spacing (i.e., spaced or massed learning condition) for each of the studies was coded and their ESs were calculated.

Firstly, the approach results showed that among the 32 studies, 93.75% of the studies was conducted in an intentional learning condition (*k* = 30), and 9.38% in an incidental learning condition (*k* = 3). An intentional learning condition was used much more often than an incidental learning condition.

Secondly, the spacing results showed that among the 26 studies, 92.31% of the studies used a spaced learning condition (*k* = 24), and 11.54% used a massed learning condition (*k* = 3), and 3.85% used both learning conditions (*k* = 1). More studies used a spaced learning condition than a massed learning condition or both conditions.

Thirdly, the effect direction plot was constructed and visually presented in Table 6. Among the 32 studies, 75% reported a positive effect (*k* = 24) and 25% reported no change (*k* = 8). There was a trend showing most studies that applied these learning conditions showed a positive effect on vocabulary learning.

**Table 6:** Effect direction plot for learning conditions (*k* = 32).

**Studies**	**Sample size (E/C)**	**Approach**	**Spacing**	**Outcomes**
Tan and Nicholson (1997)	42	Intentional learning	N/A	▴
Nakata (2008)	67/74#	Intentional learning	Spaced learning	▲
Başoglu and Akdemir (2010)	29/29	Intentional learning	Spaced learning	▴
Oberg (2011)	28/36#	Intentional learning	N/A	▴
Azabdaftari and Mozaheb (2012)	40/40#	Intentional learning	Spaced learning	▴
Komachali and Khodareza (2012)	25/25	Intentional learning	Spaced learning	▴
Kuo and Ho (2012)	30/30#	Intentional learning	Massed and spaced learning	▴
Chien (2013)	76	Intentional learning	Spaced learning	◀▶
Nikoopour and Kazemi (2014)	109	Intentional learning	Spaced learning	◀▶
Barkat and Aminafshar (2015)	15/15#	Intentional learning	Spaced learning	▴
Chien (2015)	64	Intentional learning	Spaced learning	◀▶
Hamzehbagi and Bonyadi (2015)	30/30	Intentional learning	N/A	◂▸
Lavoie (2016)	39/15	Intentional learning	Spaced learning	▴
Özer and Koçoglu (2015)	89	Incidental learning	Spaced learning	◀▶
Galedari and Basiroo (2016)	30/30	Intentional learning	Spaced learning	▴
Aminafshar (2017)	15	Intentional learning	Spaced learning	◂▸
Saputri (2017)	13	Intentional learning	Spaced learning	▴
Wu et al. (2017)	10/10	Intentional learning	Massed learning	▴
Kose and Mede (2018)	17/17	Intentional learning	Spaced learning	▴
Tsai (2018)	9/9#	Intentional learning	Massed learning	▴
Chen and Chan (2019)	48/50	Intentional learning	Spaced learning	▴
Fukushima (2019)	30	Intentional learning	Spaced learning	▴
Reynolds and Shih (2019)	100	Intentional learning	Spaced learning	▲
Alhuwaydi (2020)	42	Intentional learning	N/A	▴
Hidayat and Yulianti (2020)	27/26#	Intentional learning	Spaced learning	◂▸
Lai et al. (2020)	38/20	Intentional learning	Spaced learning	▴
Lukas et al. (2020)	52	Intentional learning	Spaced learning	▲
Reynolds et al. (2020)	50	Intentional and incidental learning	Spaced learning	▲
Samad and Makingkung (2020)	20	Intentional learning	N/A	◂▸
Wulandari and Musfiroh (2020)	34/33	Intentional learning	N/A	▴
Yüksel et al. (2020)	57	Intentional learning	Spaced learning	▲
Xodabande et al. (2021)	36/19	Intentional learning	Spaced learning	▴

E, experimental group; C, control group. # = an experimental group, i.e., the least interfering experimental group, was treated as a control group. N/A = not available in the publication or from the authors. ▴ or ▲ = positive effect, ▾ = negative effect, ◀▶ or ◂▸ = no change. Large arrow = sample size equals or >50, small arrow = sample size smaller than 50.

**Figure 5:**
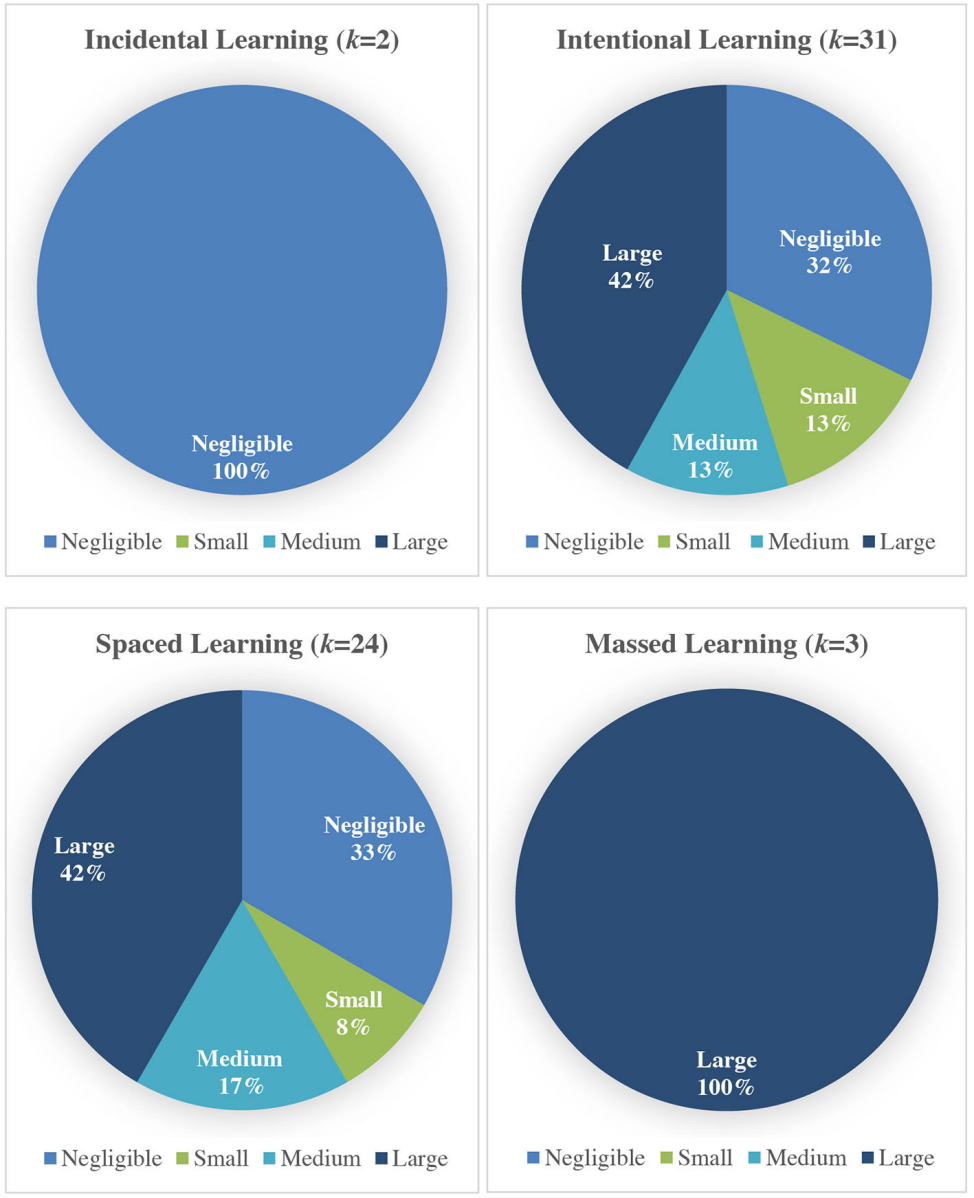
Effect size plot for learning conditions (*k* = 32).

Lastly, the effect size plot was constructed, i.e., ESs were categorized by their size and visually presented in Figure 5. In terms of the approach, the only two studies that used an incidental learning condition showed negligible effects. Studies that used an intentional learning condition showed small (13%), medium (13%) and large (42%) effects. In terms of spacing, studies that used a spaced learning condition showed small (8%), medium (17%), and large (42%) effects. All studies that used a massed learning condition showed large (100%) effects.

Based on the results of the effect size plot, more of the reviewed studies showed a larger effect for using word cards in an intentional learning condition compared with an incidental learning condition. In addition, more of the reviewed studies showed a larger effect for using word cards in a massed learning condition compared with a spaced learning condition.

### Research question 4: What is the strength of the correlation between time spent using word cards and vocabulary learning?

The fourth research question concerns the word card usage time. To examine how time spent using word cards correlates with vocabulary learning, 14 out of the 32 studies (43.75%) that provided the data on time spent using word cards by the learners and the posttest mean scores on vocabulary learning were included for analysis (Tan and Nicholson, 1997; Oberg, 2011; Kuo and Ho, 2012; Galedari and Basiroo, 2016; Wu et al., 2017; Kose and Mede, 2018; Tsai, 2018; Chen and Chan, 2019; Fukushima, 2019; Reynolds and Shih, 2019; Hidayat and Yulianti, 2020; Lukas et al., 2020; Reynolds et al., 2020; Xodabande et al., 2021).

Firstly, the time spent using word cards by the learners and the posttest mean scores on vocabulary learning were extracted from the 14 studies. Secondly, the assumptions for Spearman's Rho correlation were checked. The first assumption is that the data has to be ordinal, interval or ratio, and the second assumption is that the data has to be monotonically related, i.e., one variable increases (or decreases), the other variable also increase (or decreases) (Prion and Haerling, 2014). The extracted data is ratio, i.e., has a true or meaningful zero. In addition, the variables have a monotonic increasing relationship.

Thirdly, a Spearman's Rho correlation was run to determine the relationship between the time spent using word cards and the posttest mean scores on vocabulary learning. Spearman's rho, r_s_, for these studies were interpreted according to the following guidelines: 0 to .2 is negligible, .21 to .4 is weak, .41 to .6 is moderate, .61 to .80 is strong, and .81 to 1 is very strong (Prion and Haerling, 2014). There was a positive and weak correlation between the time spent using word cards and the vocabulary learning outcomes (*r*_*s*_=.396, *p*=.161, and *n*=14). The correlation coefficient value of .396 confirmed there was a positive and weak correlation between the two variables, meaning that both variables moved in the same direction. The *p*-value of .161 showed that there was not enough evidence to show the correlation was significant.

Based on the results of the Spearman's Rho correlation, a weak relationship was shown between time spent using word cards and vocabulary learning outcomes; however, this relationship was not found to be statistically significant.

### Research question 5: Which language proficiency group (basic, independent, or proficient) can learn the most vocabulary from using word cards?

The fifth research question concerns the proficiency level of learners that were assessed in the previous studies. To examine which proficiency group learned the most vocabulary from using word cards, 17 out of the 32 studies (53.13%) were included for analysis (Tan and Nicholson, 1997; Nakata, 2008; Başoglu and Akdemir, 2010; Oberg, 2011; Azabdaftari and Mozaheb, 2012; Komachali and Khodareza, 2012; Chien, 2013, 2015; Hamzehbagi and Bonyadi, 2015; Özer and Koçoglu, 2015; Kose and Mede, 2018; Fukushima, 2019; Reynolds and Shih, 2019; Alhuwaydi, 2020; Hidayat and Yulianti, 2020; Reynolds et al., 2020; Yüksel et al., 2020).

Firstly, the proficiency level in each of the 17 studies was coded and their ESs were calculated. Secondly, the frequency of the studies that assessed proficiency level (i.e., how many studies out of the 17 studies investigated the proficiency level) was calculated. Results showed that among the 17 studies, 47.06% involved learners at the B1 level (*k* = 8), 23.53% at the A1 level (*k* = 4), followed by B2 (*k* = 3), A2 (*k* = 1), C1 (*k* = 1), and none in the C2 (*k* = 0). Learners at the B1 level were involved more often than learners at any other proficiency level.

**Figure 6:**
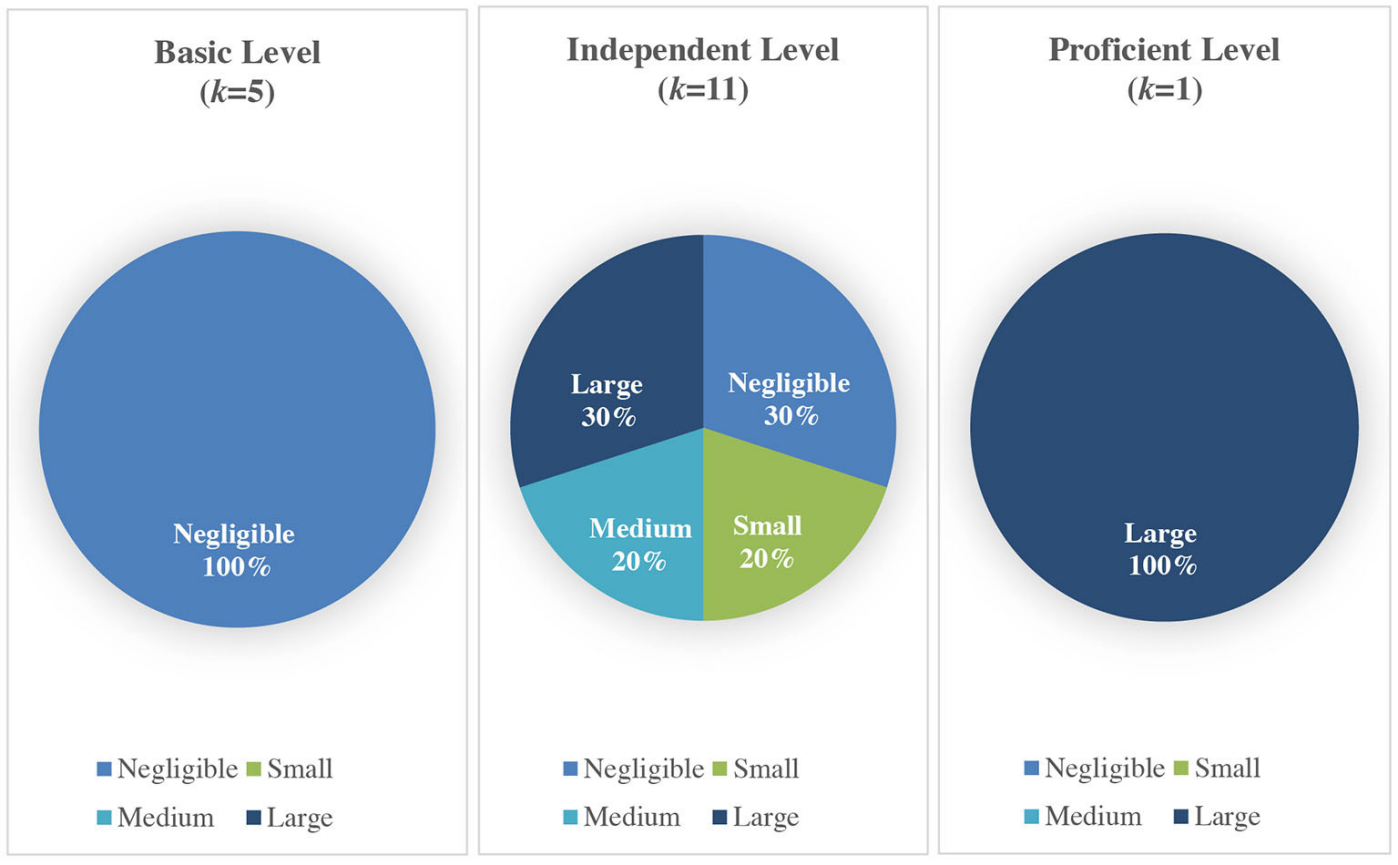
Effect size plot for proficiency level of learners (*k* = 17).

Thirdly, the effect direction plot was constructed and visually presented in Table 7. Among the 17 studies, 70.59% reported a positive effect (*k* = 12) and 29.41% reported no change (*k* = 5). There was a trend showing most studies that assessed the proficiency level of learners showed a positive effect on vocabulary learning. Lastly, the effect size plot was constructed, i.e., ESs were categorized by their size in terms of basic (i.e., A1 or A2 level), independent (i.e., B1 or B2 level) and proficient (i.e., C1 or C2 level) level group and visually presented in Figure 6. In terms of proficiency level of learners, all studies that assessed basic learners showed negligible effects. Studies that assessed independent learners showed varied effects, i.e., small (20%), medium (20%) and large (30%) effects. The 1 study that assessed proficient learners showed a large (100%) effect.

**Table 7:** Effect direction plot for proficiency level of learners (*k* = 17).

**Studies**	**Sample size (E/C)**	**Proficiency level of learners**	**Outcomes**
Tan and Nicholson (1997)	42	A1	▴
Nakata (2008)	67/74#	B1	▲
Başoglu and Akdemir (2010)	29/29	B1	▴
Oberg (2011)	28/36#	B2	▴
Azabdaftari and Mozaheb (2012)	40/40#	C1	▴
Komachali and Khodareza (2012)	25/25	B1	▴
Chien (2013)	76	B1	◀▶
Chien (2015)	64	B1	◀▶
Hamzehbagi and Bonyadi (2015)	30/30	A2	◂▸
Özer and Koçoglu (2015)	89	A1	◀▶
Kose and Mede (2018)	17/17	B1	▴
Fukushima (2019)	30	B1	▴
Reynolds and Shih (2019)	100	B2	▲
Alhuwaydi (2020)	42	B2	▴
Hidayat and Yulianti (2020)	27/26#	A1	◂▸
Reynolds et al. (2020)	50	A1	▲
Yüksel et al. (2020)	57	B1	▲

E, experimental group; C, control group. # = an experimental group, i.e., the least interfering experimental group was treated as a control group. ▴ or ▲ = positive effect, ▾ = negative effect, ◀▶ or ◂▸ = no change. Large arrow = sample size equals or >50, small arrow = sample size smaller than 50.

Based on the results of the effect size plot, learners that were more proficient in English learned more words from using word cards than those less proficient in English. More specifically, learners at the proficient level (i.e., C1 or C2 level) learned the most vocabulary from using word cards, followed by independent (i.e., B1 or B2 level) learners and finally basic (i.e., A1 or A2 level) learners.

## Discussion

### Aspects of word knowledge

The vocabulary assessments used in the reviewed studies should have assessed the aspects of word knowledge learned by the learners. As the studies included in this synthesis were related to the use of word cards, there should have been a relationship between the assessments in these studies and the use of the word cards. More specifically, Nation (1982, 2013a) suggested a simultaneous presentation of the L1 form of a target word and its meaning for the first encounter, and then a delayed presentation when using word cards. This is because a retrieval of a target word's form or meaning is necessary for learning to take place. If word cards were used to learn productive knowledge of form, i.e., learners looking at the L1 meaning and trying to recall the L2 written form, it is assumed that the productive knowledge of form would have been assessed by the researchers. Alternatively, if word cards were used to learn productive knowledge of meaning, i.e., learners looking at the L2 written form and trying to recall the L1 meaning, it is assumed that the productive knowledge of meaning would have been assessed by the researchers.

Surprisingly, researchers assessed receptive knowledge more often than productive knowledge in the reviewed studies. This might be due to two reasons. Firstly, the extent that the learners engaged in productive learning when using word cards were limited. Researchers might not have assessed productive knowledge due to learners not often using the word cards for productive learning. If word cards were used properly by the learners, the learning of productive knowledge would occur, as a retrieval of target words' forms or meanings would take place. In many of the reviewed studies, it is less certain if the learners used the word cards in this way, which could be the reason that researchers did not assess productive knowledge. Only some of the studies clearly indicated the learners used the word cards for the learning of productive knowledge (Alhuwaydi, 2020; Lukas et al., 2020; Yüksel et al., 2020).

In contrast, some other studies simply asked the learners to create word cards but gave limited instruction to the learners on how to use those word cards. For example, in Kuo and Ho's (2012) study, learners were presented with the L2 forms of the target words and were required to write the L1 meanings on the other side of the word cards. The learners were then required to share their experience of using the word card strategy in class. However, it might have been possible that the learners did not know how to use the word cards properly to do the retrievals. It should be mentioned that word card creation is not the end of the learning process. Instead, the key to word card use is for learners to look at the L2 word or L1 meaning on one side, and test themselves to see if they can recall the L1 meaning or L2 word on the other side (Komachali and Khodareza, 2012).

There were also studies that engaged learners in picture-word matching activities, but the number of retrievals were limited. For example, in Samad and Makingkung's (2020) study, learners were required to read the target words on the word cards, and then match the pictures with the target words by pasting them in task books. From what was reported in the article, it seemed the learners did not work any further with the target words. Likewise, in Oberg's (2011) study, learners were presented with L2 forms of the target words and were required to draw pictures to represent the target words on the other side of the word cards. Then they required learners to recall the L1 forms of the target words by looking at the pictures. From what was reported in the article, it appeared that was where the practice with the target words ended. However, previous research has suggested that learners should check their answers rapidly in an easy way by turning over the cards (Özer and Koçoglu, 2015). The learners should come back to the word cards repeatedly, as this process will provide them with opportunities to encounter the vocabulary (Nation, 2008; Komachali and Khodareza, 2012).

Many of the studies also seemed to indicate learners only used the word cards while they were in class. Learners should also “engage in retrieval activities” outside the classroom in their spare time (Kuo and Ho, 2012, p. 35), as word cards are convenient to carry around.

The second reason for productive knowledge being assessed less often than receptive knowledge is that productive knowledge is more difficult to assess. Although the creation of a productive knowledge test is simple, the marking of the test is potentially difficult. Usually, productive knowledge is assessed through translation (Kuo and Ho, 2012; Fukushima, 2019). However, there may be multiple possible translations for a single target word. Unlike multiple-choice tests, productive knowledge translation tests do not necessarily have a single answer. However, even with multiple possible answers, there are still reliable ways to mark a productive knowledge translation test. Nation (2013b) suggested setting a way of marking any test, i.e., the use of an answer key and a set of rules for dealing with unusual or unexpected answers should be prepared before the marking begins. The key and rules for marking could be used by anyone that is asked to mark such a test. This can provide an easy way to mark a productive knowledge test. However, if the target words are selected by the learners, which often occurs when the learners are creating their own word cards for the words selected by themselves, it may be difficult for researchers to anticipate the words selected by learners. This would prevent the researchers from being able to create a single key for marking all learners' assessment outcomes. Therefore, under the circumstances of learners constructing their own word cards, it may not be possible to create a productive knowledge test.

Vocabulary form, meaning, and use did not receive an equal amount of attention from researchers. Most researchers were interested in assessing form and meaning, possibly because the initial learning of vocabulary occurs when a form-meaning connection is made (Nation, 2013a). On the other hand, vocabulary knowledge of use is a more advanced and complex aspect of vocabulary knowledge, and many learners may “lack the opportunities or motivation to use target words” in a short term study (Yang et al., 2021, p. 479). It is less likely that vocabulary knowledge of use would have been mastered by learners and able to be assessed by researchers, that is unless the study is longitudinal (Nation, 2013a).

Researchers used standardized tests or researcher-constructed tests as vocabulary measurements. If learners' general vocabulary knowledge was to be assessed, standardized tests were used by the researchers. Alternatively, if learners' knowledge of specific lexical items were to be assessed, researchers constructed special tests for this purpose. It should be mentioned that most standardized tests used in the previous studies measured the breadth of vocabulary knowledge, i.e., “the vocabulary size of learners” (Azabdaftari and Mozaheb, 2012, p. 48). It is not surprising that the main standardized tests that were used in the reviewed studies, i.e., VST (Nation and Beglar, 2007), UVLT (Webb et al., 2017) and NGSLT (Browne et al., 2013), were all designed to assess learners' receptive knowledge of meaning.

These tests were found to possess the characteristics of reliability, validity and practicality, which are the three major characteristics of a good test (Nation, 2013b). These tests were a reliable measure of vocabulary size due to their adequate sampling of vocabulary items. These tests were valid because they were measuring what they were supposed to measure, i.e., a vocabulary size test should measure learners' vocabulary size. These tests were practical because they were easy to administer (a computer program can be used), easy to mark (layout of the tests facilitates marking) and easy to interpret (tested words represent the whole population of words from which they were chosen) (Nation, 2013b).

Considering the characteristics of reliability, validity and practicality can also help to explain the researcher-constructed tests used in the reviewed studies. More specifically, receptive knowledge multiple-choice tests are often very practical in terms of marking but could reduce validity, as it should be more valid for a learner to provide an answer than to choose from a range of choices (Nation, 2013b). For example, Oberg (2011) assessed receptive knowledge by having learners take a sentence fill-in-the-blank test with multiple-choice items given. On the other hand, productive tests are often valid but somehow not practical, as this test format might be challenging to mark. For example, Özer and Koçoglu (2015) assessed productive knowledge by having learners write a composition using target words. Even with the reduction of practicality, it is necessary to assess learners' productive knowledge gains from the use of word cards. This is because practicality is not as important as reliability and validity in a test (Nation, 2013b). However, this lack of practicality could also be the reason that less productive knowledge tests were used in previous research on word card use.

### Types of word cards

Interestingly, more of the reviewed studies showed a larger effect for the use of paper word cards compared to digital word cards. It should be mentioned that the comparison between paper word cards and digital word cards was an indirect comparison by looking at the ESs of the particular word card type used in the studies. More studies showed a larger effect for the use of paper word cards compared to digital word cards for two reasons. Firstly, the use of paper word cards might be more suitable for young learners (Azabdaftari and Mozaheb, 2012). Paper word cards allow for easier interaction between learners (Komachali and Khodareza, 2012) and do not necessitate the learning of a computer program to use them (Reynolds et al., 2020). The familiarization with digital word cards requires time and energy, so teachers or technical staff may be needed to assist the learners to use digital word cards (Azabdaftari and Mozaheb, 2012). Secondly, most studies compared the use of paper word cards to other vocabulary learning activities, i.e., gesture-based systems (Wu et al., 2017), wordlists (Kuo and Ho, 2012) or a control group without any intervention (Komachali and Khodareza, 2012), rather than comparing the two types of word cards. As the use of paper word cards and other activities mentioned above were quite different, it is not surprising that paper word cards were found to be more effective than these other activities. Therefore, the results of this synthesis which indicated paper word cards were more effective than digital word cards should be considered with caution.

Certain studies compared the use of digital word cards and paper word cards. They showed no significant difference in the effectiveness of these two types of word cards (Oberg, 2011; Nikoopour and Kazemi, 2014; Chen and Chan, 2019). However, some studies showed digital word cards had a larger effect than paper word cards on vocabulary learning (Başoglu and Akdemir, 2010; Azabdaftari and Mozaheb, 2012). Unlike the studies that investigated paper word cards in comparison to other activities, these studies compared digital word cards to paper word cards. Under this circumstance, the use of paper word cards and digital word cards were two similar types of activities. Therefore, it is not surprising that there was a non-significant difference between the use of paper word cards and digital word cards. However, there still were some studies that showed a larger effect with the use of digital word cards. For these studies, the additional affordances of digital media such as incorporating sounds (Başoglu and Akdemir, 2010; Barkat and Aminafshar, 2015; Fukushima, 2019), animations (Barkat and Aminafshar, 2015; Chen and Chan, 2019) and videos (Chen and Chan, 2019) could have been the reason for the better learning outcome, even though previous researchers have suggested that these could be distractions for learners (Chen and Chan, 2019).

More of the reviewed studies showed a larger effect for the use of ready-made cards than self-constructed word cards. Teachers are usually aware of their learners' proficiencies and could select target words at an appropriate level of difficulty for their learners. It is important for learners to focus on learning vocabulary that is at the right level of difficulty. Learners should focus on learning the most frequent words in a language first (Nation, 2013a). In other words, the first 1,000 words should be learned before the second 1,000 words, and the second 1,000 words should be learned before the third 1,000 words, and so on (Nation, 2013a). In this regard, if a learner had not mastered the first 1,000 most frequent words of English, the learner should not try to learn words from the third 1,000 most frequent words of English.

Teachers are usually aware of the proficiency level of their learners, so they may have been in a better situation to select the most appropriate target words for learners (Read, 2000). On the other hand, the self-constructed word cards that were created by the learners on their own may have contained target words that were not at the appropriate level, i.e., the words could have been too easy or too difficult for learners. It could have been that the learners in the previous studies were not well-equipped at target word selection. Teachers should give guidance and training on how to select target words that are appropriate for learners, because the most frequent words of English need to be mastered first (Nation, 2013a). It is difficult to know exactly how the learners used the word cards in the previous research, as some studies only explained the steps involved in word card construction (e.g., Chien, 2013) or how the word cards were used (e.g., Kose and Mede, 2018), but not both. Overall, it is difficult to determine the difference in the effectiveness of ready-made word cards and self-constructed word cards, as none of the included studies compared these two types of word cards.

### Use of word cards

#### Learning conditions

More of the reviewed studies showed a larger effect for using word cards in an intentional learning condition compared with an incidental learning condition. However, only 2 of the 32 studies used an incidental learning condition (Özer and Koçoglu, 2015; Reynolds et al., 2020). Therefore, it is premature to conclude that the intentional learning condition would benefit vocabulary learning more than the incidental learning condition when using word cards. It is not surprising that most of the studies used an intentional learning condition as the use of word cards is an intentional learning strategy (Nation, 2013a). Unless the use of the word cards was manipulated by the researchers to create an experiment in incidental learning, it was less likely that use of word card could result in incidental learning.

Although word cards are usually an intentional learning strategy, they can be used for incidental learning purposes. This is important because intentional and incidental learning should complement each other (Nation, 2013a). For example, a learner who reads an article incidentally could come across an unknown word and then record that word on a word card for later review (Reynolds et al., 2020). Except for the first few thousand most common words of English, most vocabulary should be learned incidentally (Lin and Lin, 2019). After a mastery of these most frequent words using intentional learning strategies, the learner can work on increasing their vocabulary size with incidental vocabulary learning strategies (Lin and Lin, 2019).

Interestingly, more of the reviewed studies showed a larger effect for using word cards in a massed learning condition compared with a spaced learning condition. A teacher who trains learners on how to use word cards usually tells the learners to use the word cards in a spaced learning condition (Kuo and Ho, 2012). However, previous research has suggested that when new words are first introduced to learners, a massed learning condition may be more effective (Uchihara et al., 2019). During this initial learning, learners should work with the word cards in a massed learning condition because that will result in a large number of repeated encounters with the words (Uchihara et al., 2019).

There were still certain studies that found spaced learning led to better but non-significant differences than massed learning (Kuo and Ho, 2012). Thus, spaced learning could still potentially be more effective than massed learning. However, it could be that learners should use massed learning initially and then follow up with spaced learning. Moreover, previous researchers have suggested to increasingly spread out the meetings with newly learned words using a distribution schedule where the repetitions become increasingly further apart (Nation, 2008). Revisiting of previously learned words can strengthen retention of vocabulary knowledge (Nation, 2008).

#### Time spent learning form word cards

A positive non-significant weak correlation was found between the time that learners spent using word cards and their posttest vocabulary scores. The positive relationship suggested that the more time learners spent on vocabulary learning from word cards, the more vocabulary they learned. There might be two reasons for the statistically insignificant result. Firstly, there were only 14 studies that provided necessary data that could be extracted for analysis in this synthesis. The relatively small sample size might increase variability, which resulted in a statistically non-significant correlation. Secondly, the lack of a meaningful relationship between the learning time and vocabulary learning may be due to the “limited ability of certain learners to learn effectively” from word cards (Nakata, 2008, p. 3). Even though certain learners spent more time, if they did not use the time efficiently, they might not learn no matter how much time they spent.

Although the correlation was not statistically significant, there is research that has indicated more time spent on using word cards results in more vocabulary learning (Webb et al., 2020). It should be mentioned that what is more important than the overall amount of time that learners spend using the word cards is probably how they use those word cards. For example, learners should be repeatedly coming back to words instead of meeting them all at once, which is often referred to as spaced learning (Nation and Webb, 2011; Nation, 2013a).

Another issue that was unclear in the previous studies was whether the words that the learners worked on were semantically related or not. Previous researchers showed that when learners worked with a new group of words that are semantically related to each other, it could be more difficult to acquire them rather than if these words were not semantically related to each other (Tinkham, 1997; Nation and Webb, 2011). When learners are trying to learn a set of semantically related words, they will confuse words that are too similar, which could increase the learning difficulty (McDonald and Reynolds, 2021). In contrast, if the words are not related to each other or organized thematically, it may lead to a better learning outcome. This is because differences between lexical items facilitates learning (McDonald and Reynolds, 2021).

### Learner proficiency

Learners at the proficient level (i.e., C1 or C2 level) learned the most vocabulary from using word cards, followed by independent (i.e., B1 or B2 level) learners and finally basic (i.e., A1 or A2 level) learners. In other words, learners that were more proficient in English learned more words from using word cards than those less proficient in English. There is a possibility that learner proficiency has a moderating effect on vocabulary learning. Previous researchers have indicated that vocabulary development progresses differently for learners at different proficiency levels (Elgort, 2017). However, it is a relatively under-researched area in the word card literature, as only one of the included studies involved learners at different proficiency levels in a single study (Tsai, 2018). Tsai (2018) found that the learners at the higher proficiency level had more effective learning outcomes than learners at the lower proficiency level.

Language proficiency could be related to the amount of effort needed to invest in the learning task. Learners with lower proficiencies might have to work very hard to learn, which they might consider as a time-consuming task (Elgort, 2017). However, it might be easier for learners with higher proficiencies to gain more vocabulary knowledge, so they might be more willing to invest more time in learning. More proficient learners have more autonomy to take better charge of their learning (Lin and Lin, 2019), and therefore may be more skillful in vocabulary learning using word cards (de Vos et al., 2018). For example, in a study conducted by Azabdaftari and Mozaheb (2012), proficient learners used the word cards both inside and outside the classroom. This may have allowed them to devote more attention to unknown words and may have increased the potential for vocabulary learning. Learners with lower proficiency might stop using the word cards when class is over, even if they are encouraged to use word cards outside of class.

## Conclusion

### Limitations

Although this synthesis uncovered some interesting findings, some limitations must be discussed. Firstly, a literature search was only conducted in Scopus and WOS (including SCI-Expanded, SSCI, AHCI, CPCI-S, CPCI-SSH, BKCI-S, BKCI-SSH, ESCI). Thus, the coverage of the synthesis is limited to these databases.

Secondly, this synthesis only included published research. Peer-reviewed studies were selected for review to ensure quality, but this opens up the possibility of publication bias.

Thirdly, a synthesis method was adopted to visually present the effect sizes. The studies included in the synthesis were not implemented with totally identical research designs and did not assess the same aspect of word knowledge. Although ESs were calculated for each included study, we did not look at the significant differences between moderating variables. Instead, the effect direction plots and effect size plots (ESs were categorized by their size) were presented. We took this approach which was different from what would be done with meta-analyses in order to be able to include more primary studies in this synthesis.

Finally, only the immediate posttest data was used for ESs analysis. We only looked at the immediate posttest data because all primary studies provided this data. Therefore, the long-term effects of vocabulary learning from word cards were not investigated in this synthesis. With these limitations in mind, the research implications and teaching implications of the current study results are reported below.

### Research implications

One suggestion for future research is that learner proficiency should be reported. Fifteen of the 32 included studies (46.88%) did not report the proficiency level of the learners. It is difficult to interpret the results of studies that do not clearly describe learner proficiency. Due to the uncertainty of learner proficiency, it is also difficult to conclude whether the vocabulary learning reported in such studies can be generalized to certain learner populations. In addition, learners at different proficiency levels can be recruited for future studies. These studies could compare the effects of word card use on basic, independent, and proficient learners' vocabulary learning.

Another suggestion for future research is that appropriate tests should be adopted to test vocabulary learning performance. For example, future researchers can adopt more standardized tests or report the reliability for researcher-constructed tests. Since most of the included studies that used researcher-constructed tests did not provide any reliability measures for the tests, the effects of the word card intervention reported in the studies is questionable. In addition, productive tests should be used for testing learners' vocabulary knowledge gained from the use of word cards, as productive knowledge production should have taken place during word card use.

Future researchers can also compare the effectiveness of ready-made word cards and self-constructed word cards, as none of the reviewed studies compared the effect of these two types of word cards. In other words, the effects of both types of word cards had been studied separately but were not compared in a single study.

Another interesting area for future research may be to investigate the learning of different aspects of word knowledge. In this synthesis, most studies started with receptive knowledge in their vocabulary assessments. To prevent practice effects, i.e., previous tests could affect subsequent test performance, so the scores of the first receptive knowledge assessment in each study were extracted for analysis. This synthesis provides a certain understanding of how receptive knowledge of form and meaning can be acquired through the use of word cards. However, we are less certain of the effects of the variables in the current synthesis have on other aspects of word knowledge acquired through the use of word cards. As more time may be needed to develop vocabulary knowledge of use, future longitudinal studies can be conducted to address this gap in the literature.

### Teaching implications

A teacher that decides to incorporate the use of word cards inside or outside their language classroom should take the following into consideration. Whether using digital or paper word cards, teachers should spend adequate time providing guidance to learners on how to use word cards properly (Chen and Chan, 2019). Some digital programs could offer teachers some affordances such as ready-made word banks and streamlined use of the word cards. If a teacher chooses to use digital word cards, large screen tablets or computer should be used, because they can provide a better learning experience. While the synthesis did not aim to investigate how to use digital word cards for better learning outcomes, teachers who plan to use digital word cards should consider the screen size in a computer program or a mobile app. This is because previous researchers (Ji and Aziz, 2021) indicated that learners might have difficulty leaning vocabulary on devices with limited screen sizes.

For some learners, self-constructed word cards can save teacher planning time (Reynolds et al., 2020), but require proper guidance before learners begin constructing the word cards (Reynolds and Shih, 2019) and additional checks afterwards by teachers (Reynolds et al., 2020). For self-constructed word cards, teachers should guide the learners in target word selection, especially for those who have just received training on how to use word cards.

A teacher should consider encouraging different learning conditions when learners are using word cards. Intentional learning of vocabulary using word cards is very effective, but teachers can also consider asking learners to use word cards for new words they have incidentally encountered through engagement in other non-language learning tasks, such as reading, watching videos (Lin and Lin, 2019), or classroom discussion (Uchihara et al., 2019). Teachers should stress the importance of when spaced and massed learning should be applied to word card use. Teachers can encourage learners to use massed learning when they work with new words at the beginning, and use spaced learning later on. Learners also need to be reminded to use the word cards frequently throughout the day. Many of these guidelines for using word cards have also been incorporated into certain digital apps. However, if a teacher suggests digital apps to learners, the teacher should make sure the digital apps possess these qualities before recommending them to learners.

## Data availability statement

The original contributions presented in the study are included in the article/Supplementary material, further inquiries can be directed to the corresponding author/s.

## Author contributions

BR contributed to the conception and design of the study and revised the paper. YL collected, organized, analyzed the data, and drafted the paper. BR and YL interpreted the results, approved the submitted version of the paper, responded to the reviewer comments, and revised the submitted version of the paper. Both authors contributed to the article and approved the submitted version.

## Funding

This research was supported by the University of Macau under Grant Number MYRG2019-00030-FED.

## Conflict of interest

The authors declare that the research was conducted in the absence of any commercial or financial relationships that could be construed as a potential conflict of interest.

## Publisher's note

All claims expressed in this article are solely those of the authors and do not necessarily represent those of their affiliated organizations, or those of the publisher, the editors and the reviewers. Any product that may be evaluated in this article, or claim that may be made by its manufacturer, is not guaranteed or endorsed by the publisher.
