# Supplementary material for: Learning English vocabulary from word cards: A research synthesis
Source: Front Psychol. 2022 Sep 6;13:984211. doi: 10.3389/fpsyg.2022.984211 (PMC9485613; doi:10.3389/fpsyg.2022.984211)
Supplement: Supplementary file 1 [file Table_1.DOCX]

Supplementary Material

Table S1

*Coding Scheme*

| **Features** | **Description** |
| --- | --- |
| ***Bibliographic information*** |  |
| Author(s) | Researchers who conducted and published the study |
| Title | Title of the study |
| Year of publication | The year in which the study was published |
| Type of publication | Type of the publication (i.e., journal article, book chapter, conference paper) |
| Country or region | Country or region where the study was conducted (e.g., Malaysia, Indonesia, Japan, Turkey, etc.) |
| ***Learner characteristics*** |  |
| Sample size | Number of learners |
| Proficiency level | The Common European Framework of Reference for Languages (CEFR) (Council of Europe, 2001) is “the most influential language framework in the field of second language teaching and assessment” (Fleckenstein et al., 2020, p. 2). “It describes foreign language competencies in three broad stages which can be divided into six proficiency levels”, i.e., A1/A2 for basic users, B1/B2 for independent users, and C1/C2 for proficient users (Fleckenstein et al., 2020, p. 2). |
| Educational level | Educational level of learners (i.e., preprimary, primary, secondary, university) |
| L1 background | First language of learners |
| ***Word card characteristics*** |  |
| Number of target words | Number of words available to participants for learning |
| Origin of word cards | Origin of word cards refers to the producers of word cards. They can be the learners (Self-constructed), teachers (Ready-made), or both learners and teachers (Both). |
| Digital integration | Digital integration refers to the word cards not containing any digital integration (None), containing digital integration through the use of a computer program (Computer program), or containing digital integration through the use of a mobile app (Mobile app). |
| Vocabulary learning time (days) | Number of days the learners spent learning vocabulary using word cards |
| Word card usage time (minutes) | Number of minutes the learners spent learning vocabulary using word cards |
| Semantic relatedness | Semantic relatedness refers to the vocabulary studied with the word cards organized into *semantic clusters*, “sets of semantically and syntactically similar words, e.g., eye, nose, ear, mouth, chin” (Semantic) (Tinkham, 1997, p. 138), or organized thematically, “e.g., frog, green, hop, pond, slippery, croak” (Thematic) (Tinkham, 1997, p. 138). |
| Type of assessed vocabulary | Type of assessed vocabulary refers to learners being assessed on specific vocabulary knowledge (Specific vocabulary) or general vocabulary knowledge (General vocabulary). |
| Type of vocabulary test | Type of vocabulary test refers to whether the vocabulary test in the study was a researcher-constructed test or a standardized test. The three standardized tests used were The Vocabulary Size Test (VST) (Nation & Beglar, 2007), The Updated Vocabulary Levels Test (UVLT) (Webb et al., 2017), and New General Service Lists Test (NGSLT) (Browne et al., 2013). |
| ***Methodological characteristics*** |  |
| Study design | Study design refers to the research type used for the study. This included the independent-groups posttest design in which the outcome was measured “at a single point in time and was compared across independent groups that receive different treatments” (e.g., experimental and control groups) (Independent-groups posttest) (Morris & DeShon, 2002, p. 107), the single-group pretest-posttest design in which “all learners received the same treatment, and scores on the outcome were compared before and after treatment was administered” (Single-group pretest-posttest) (Morris & DeShon, 2002, pp. 107-108), the independent-groups pretest-posttest design in which “the outcome was measured before and after treatment, and different groups received different treatments” (e.g., experimental and control groups) (Independent-groups pretest-posttest) (Morris & DeShon, 2002, pp. 107-108), or other study design (e.g., qualitative study in which no statistical information was given) (Others). |
| Theoretical perspective | Theoretical perspectives used were the Involvement Load Hypothesis (Laufer & Hulstijn, 2001), Pimsleur’s Memory Schedule (Pimsleur, 1967), and Dual-Coding Theory (Paivio, 1979). |
| Control group | Control group refers to the absence (None) or presence (Yes) of a control group, i.e., the group without using word cards or other interventions in the study. |
| Pretest | Pretest refers to the absence (None) or presence (Yes) of a pretest in the study. |
| Test timing | Test timing refers to whether an immediate posttest (Immediate posttest), delayed posttest (Delayed posttest), or both (Both) were used in the study. |
| ***Learning condition*** |  |
| Approach | Approach refers to either incidental learning where learners are “unaware of a subsequent vocabulary test” (Incidental learning), or intentional learning where they are aware of a subsequent vocabulary test (Intentional learning) (Webb et al., 2020, p. 2). |
| Spacing | Spacing refers to whether the word cards were used in either a massed learning condition or a spaced learning condition. Massed learning refers to use of word cards that were completed within a single day, i.e., the study was conducted in a massed learning condition (Massed learning) (Uchihara et al., 2019), and spaced learning refers to use of word cards that lasted for more than one day, i.e., the study was conducted in a spaced learning condition (Spaced learning) (Uchihara et al., 2019). |
| ***Aspects of word knowledge*** |  |
| Receptive form | Receptive form refers to whether the learner can “recognize the spoken form of the word, written form of the word, or the known parts in the word” (Nation, 2013a, p. 538). |
| Productive form | Productive form refers to whether the learner can “pronounce the word correctly, spell and write the word, or produce appropriate inflected and derived forms of the word” (Nation, 2013a, p. 538). |
| Receptive meaning | Receptive meaning refers to whether the learner can “recall the appropriate meaning for the word form, understand a range of uses of the word and its central concept, or recall common associations for the word” (Nation, 2013a, p. 538). |
| Productive meaning | Productive meaning refers to whether the learner can “produce the appropriate word form to express the meaning, use the word to refer to a range of items, or recall the word when presented with related ideas” (Nation, 2013a, p. 538). |
| Receptive use | Receptive use refers to whether the learner can “recognize correct uses of the word in context, recognize appropriate collocations, or tell if the word is a common, formal, or infrequent word” (Nation, 2013a, p. 538). |
| Productive use | Productive use refers to whether the learner can “use the word in correct grammatical patterns, produce the word with appropriate collocations, or use the word at appropriate times” (Nation, 2013a, p. 538). |
| ***Results*** |  |
| Mean_post,E_ | Mean of experimental group posttest scores |
| Mean_pre,E_ | Mean of experimental group pretest scores |
| Mean_post,C_ | Mean of control group posttest scores (independent-groups pretest-posttest design only) |
| Mean_pre,C_ | Mean of control group pretest scores (independent-groups pretest-posttest design only) |
| SD_post,E_ | Standard deviation of experimental group posttest scores |
| SD_pre,E_ | Standard deviation of experimental group pretest scores |
| SD_post,C_ | Standard deviation of control group posttest scores (independent-groups pretest-posttest design only) |
| SD_pre,C_ | Standard deviation of control group pretest scores (independent-groups pretest-posttest design only) |

Table S2

*Overview of the 32 Included Studies*

| **Bibliographic Information** | | **Learner Characteristics** | | **Word Card Characteristics** | | | | **Learning Conditions** | | **Aspects of Word Knowledge** | **ESs** | **RQs** |
| --- | --- | --- | --- | --- | --- | --- | --- | --- | --- | --- | --- | --- |
| ***Studies*** | ***Type of publication*** | ***N***  ***(E/C)*** | ***Proficiency level*** | ***No. of target words*** | ***Origin of word cards*** | ***Digital integration*** | ***Time (mins)*** | ***Approach*** | ***Spacing*** |  |  |  |
| Tan and Nicholson (1997) | Journal article | 42 | A1 | N/A | Ready-made | None | 100 | Intentional learning | N/A | RF, RU | 0.07③ | 1, 2, 3, 4, 5 |
| Nakata (2008) | Journal article | 67/74# | B1 | 10 | Ready-made | Computer program | N/A | Intentional learning | Spaced learning | RM, PM | 0.06③ | 1, 2, 3, 5 |
| Başoğlu and Akdemi̇r (2010) | Journal article | 29/29 | B1 | 1000 | Ready-made | Mobile app | N/A | Intentional learning | Spaced learning | RF | 0.37② | 1, 2, 3, 5 |
| Oberg (2011) | Journal article | 28/36# | B2 | 10 | Ready-made | Computer program | 100 | Intentional learning | N/A | RM | 0.27② | 1, 2, 3, 4, 5 |
| Azabdaftari and Mozaheb (2012) | Journal article | 40/40# | C1 | N/A | Ready-made | Mobile app | N/A | Intentional learning | Spaced learning | RM | 1.30③ | 1, 2, 3, 5 |
| Komachali and Khodareza (2012) | Journal article | 25/25 | B1 | 60 | Ready-made | None | N/A | Intentional learning | Spaced learning | PM | 2.59② | 1, 2, 3, 5 |
| Kuo and Ho (2012) | Journal article | 30/30# | N/A | 120 | Ready-made | None | 120 | Intentional learning | Both | RM, PM | 2.18② | 1, 2, 3, 4 |
| Chien (2013) | Conference paper | 76 | B1 | N/A | Self-constructed | Computer program | 1920 | Intentional learning | Spaced learning | RF, PF | 0† | 1, 2, 3, 5 |
| Nikoopour and Kazemi (2014) | Journal article | 109 | N/A | 700 | Ready-made | Mobile app | N/A | Intentional learning | Spaced learning | RM | 0† | 1, 2, 3 |
| Barkat and Aminafshar (2015) | Journal article | 15/15# | N/A | N/A | Ready-made | Computer program | N/A | Intentional learning | Spaced learning | RM | 3.18② | 1, 2, 3 |
| Chien (2015) | Journal article | 64 | B1 | N/A | Self-constructed | Computer program | 1440 | Intentional learning | Spaced learning | RF, PF | 0† | 1, 2, 3, 5 |
| Hamzehbagi and Bonyadi (2015) | Journal article | 30/30 | A2 | 40 | Ready-made | None | N/A | Intentional learning | N/A | RM | 0† | 1, 2, 3, 5 |
| Lavoie (2016) | Journal article | 39/15 | N/A | 15 | Ready-made | None | N/A | Intentional learning | Spaced learning | RM | 2.70② | 1, 2, 3 |
| Özer and Koçoğlu (2015) | Conference paper | 89 | A1 | 123 | Ready-made | Computer program | N/A | Incidental learning | Spaced learning | RF, PF, RM, PM, RU, PU | 0† | 1, 2, 3, 5 |
| Galedari and Basiroo (2016) | Journal article | 30/30 | N/A | 20 | Ready-made | None | 7200 | Intentional learning | Spaced learning | RF, PF, RM, PM | 2.88③ | 1, 2, 3, 4 |
| Aminafshar (2017) | Journal article | 15 | N/A | N/A | Ready-made | Computer program | N/A | Intentional learning | Spaced learning | RF | 0† | 1, 2, 3 |
| Saputri (2017) | Conference paper | 13 | N/A | N/A | Ready-made | None | N/A | Intentional learning | Spaced learning | RF | 0.52① | 1, 2, 3 |
| Wu et al. (2017) | Book chapter | 10/10 | N/A | 10 | Ready-made | None | 4 | Intentional learning | Massed learning | RF, RM | 2.74② | 1, 2, 3, 4 |
| Kose and Mede (2018) | Journal article | 17/17 | B1* | 90 | Self-constructed | Mobile app | 1500 | Intentional learning | Spaced learning | N/A | 1.85② | 2, 3, 4, 5 |
| Tsai (2018) | Journal article | 9/9# | N/A | 20 | Ready-made | Mobile app | 30 | Intentional learning | Massed learning | N/A | 1.13③ | 2, 3, 4 |
| Chen and Chan (2019) | Journal article | 48/50 | N/A | 40 | Ready-made | Computer program | 140 | Intentional learning | Spaced learning | RM | 0.31② | 1, 2, 3, 4 |
| Fukushima (2019) | Journal article | 30 | B1 | 20 | Ready-made | Mobile app | 10 | Intentional learning | Spaced learning | RM, PM | 0.60① | 1, 2, 3, 4, 5 |
| Reynolds and Shih (2019) | Journal article | 100 | B2 | 100 | Self-constructed | None | 8640 | Intentional learning | Spaced learning | RM | 0.54② | 1, 2, 3, 4, 5 |
| Alhuwaydi (2020) | Journal article | 42 | B2* | 60 | Self-constructed | Mobile app | N/A | Intentional learning | N/A | PF, RM, RU, PU | 1.40① | 1, 2, 3, 5 |
| Hidayat and Yulianti (2020) | Journal article | 27/26# | A1* | 10* | Ready-made | Computer program | 5* | Intentional learning | Spaced learning | RF*, PF*, RM* | 0.00② | 1, 2, 3, 4, 5 |
| Lai et al. (2020) | Journal article | 38/20 | N/A | N/A | Ready-made | Mobile app | N/A | Intentional learning | Spaced learning | N/A | 0.60② | 2, 3 |
| Lukas et al. (2020) | Journal article | 52 | N/A | 20 | Ready-made | None | 300 | Intentional learning | Spaced learning | RF, PF, RM | 1.70① | 1, 2, 3, 4 |
| Reynolds et al. (2020) | Journal article | 50 | A1 | 100 | Self-constructed | None | 4320 | Intentional & incidental learning | Spaced learning | RM | 0.14① | 1, 2, 3, 4, 5 |
| Samad and Makingkung (2020) | Conference paper | 20 | N/A | N/A | Ready-made | None | N/A | Intentional learning | N/A | PM, RM | 0† | 1, 2, 3 |
| Wulandari and Musfiroh (2020) | Journal article | 34/33 | N/A | N/A | Ready-made | None | N/A | Intentional learning | N/A | RF, RM | 0.44② | 1, 2, 3 |
| Yüksel et al. (2020) | Journal article | 57 | B1 | 240 | Ready-made | Computer program | N/A | Intentional learning | Spaced learning | RM | 1.51③ | 1, 2, 3, 5 |
| Xodabande et al. (2021) | Journal article | 36/19 | N/A | 1801 | Ready-made | None & Mobile app | 7800 | Intentional learning | Spaced learning | RF, PF, RM | 2.37② | 1, 2, 3, 4 |

*Note.* *=data provided by the author(s). N/A=not available in the publication or from the authors. E=experimental group, C=control group. #= an experimental group, i.e., the least interfering experimental group, was treated as a control group. RF=receptive knowledge of form, PF=productive knowledge of form, RM=receptive knowledge of meaning, PM=productive knowledge of meaning, RU=receptive knowledge of use, PU=productive knowledge of use. ①=ESs calculated with formula 1 from Results, ②=ESs calculated with formula 2 from Results, ③=ESs calculated with formula 3 from Results. †= The conservative estimates of ESs were filled in for the studies that had missing data, i.e., assigning ESs of zero. RQs=the research questions that were answered with the data extracted from the studies.
